# miR-34/449 control apical actin network formation during multiciliogenesis through small GTPase pathways

**DOI:** 10.1038/ncomms9386

**Published:** 2015-09-18

**Authors:** Benoît Chevalier, Anna Adamiok, Olivier Mercey, Diego R. Revinski, Laure-Emmanuelle Zaragosi, Andrea Pasini, Laurent Kodjabachian, Pascal Barbry, Brice Marcet

**Affiliations:** 1 CNRS, Institut de Pharmacologie Moléculaire et Cellulaire (IPMC), UMR-7275, 660 route des Lucioles, 06560 Sophia-Antipolis, France; 2 University of Nice-Sophia-Antipolis (UNS), Institut de Pharmacologie Moléculaire et Cellulaire, 660 route des Lucioles, Valbonne, 06560 Sophia-Antipolis, France; 3 Aix-Marseille Université, CNRS, UMR7288, Institut de Biologie du Développement de Marseille (IBDM), 13288 Marseille, France

## Abstract

Vertebrate multiciliated cells (MCCs) contribute to fluid propulsion in several biological processes. We previously showed that microRNAs of the miR-34/449 family trigger MCC differentiation by repressing cell cycle genes and the Notch pathway. Here, using human and *Xenopus* MCCs, we show that beyond this initial step, miR-34/449 later promote the assembly of an apical actin network, required for proper basal bodies anchoring. Identification of miR-34/449 targets related to small GTPase pathways led us to characterize R-Ras as a key regulator of this process. Protection of *RRAS* messenger RNA against miR-34/449 binding impairs actin cap formation and multiciliogenesis, despite a still active RhoA. We propose that miR-34/449 also promote relocalization of the actin binding protein Filamin-A, a known *RRAS* interactor, near basal bodies in MCCs. Our study illustrates the intricate role played by miR-34/449 in coordinating several steps of a complex differentiation programme by regulating distinct signalling pathways.

## Abstract (web summary)

MicroRNAs of the miR-34/449 family initiate formation of multiciliated cells through the suppression of cell cycle genes and Notch. Here the authors show that miR-34/449 also regulate the assembly of an apical actin network necessary for basal body anchoring by regulating the expression of R-Ras.

## Graphical abstract

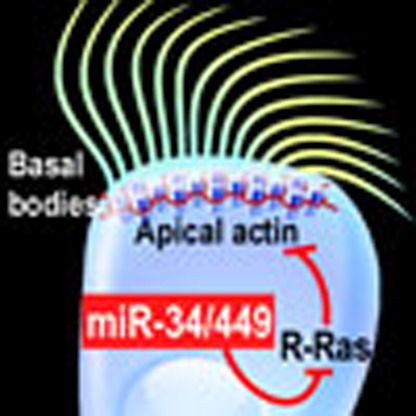

Multiciliated cells (MCCs), characterized by the presence of multiple motile cilia at their apical surface, have been described in many vertebrates^1^,^2^. Coordinated ciliary beating allows efficient fluid movement and is required for physiological processes such as elimination of mucus from the respiratory tract, circulation of the cerebrospinal fluid or migration of the embryo in the fallopian tubes^1^. The physiological importance of MCCs is highlighted by the ever growing number of human disorders associated with defects of the motile cilia^1^,^3^,^4^,^5^. Multiciliogenesis, which occurs during normal development and during regeneration of damaged tissues, can be studied in experimental setups, such as primary cultures of human airway epithelium^6^ and *Xenopus* embryonic epidermis^7^. Several characteristic steps are observed as follows: (i) exit from the cell cycle of MCC precursors, (ii) massive postmitotic multiplication of centrioles (centriologenesis), (iii) reorganization of the apical actin cytoskeleton into a dense cortical meshwork of actin, (iv) migration of the newly synthesized centrioles towards the apical pole of the cell, where they anchor to the actin meshwork and mature into ciliary organizing centres known as basal bodies, and (v) elongation of one cilium from each basal body^8^,^9^,^10^,^11^,^12^,^13^,^14^,^15^. Several key regulators of multiciliogenesis have been identified, such as Notch and bone morphogenetic protein (BMP) pathways^16^,^17^, the transcription factors FOXJ1, MYB and RFXs (regulatory factor X)^18^,^19^,^20^,^21^,^22^,^23^,^24^, and the geminin-related nuclear protein Multicilin^25^. During multiciliogenesis, the reorganization of the apical actin cytoskeleton is controlled by several factors including FOXJ1, Multicilin, the ERK7 mitogen-activated protein kinase and small GTPases such as RhoA^14^,^19^,^20^,^25^,^26^,^27^,^28^. Following FOXJ1- and RhoA-pathway-dependent phosphorylation, proteins of the ezrin-radixin-moesin (ERM) family, which link actin to the cell membrane, can interact with cortical actin^29^,^30^. The subcellular localization of ezrin and its interacting protein EBP50 at the apical membrane of airway MCCs also appears to be mediated by a FOXJ1-dependent mechanism^14^,^19^,^31^,^32^. Focal adhesion proteins are also required for the interaction between basal bodies and apical actin network during multiciliogenesis^33^. The action of small GTPases on actin cytoskeletal dynamics is regulated by a complex network of interactions with additional GTPases, such as the Ras family member R-Ras^34^,^35^,^36^,^37^,^38^,^39^, and other regulatory factors including guanine nucleotide exchange factors, GTPase-activating proteins (GAPs), GDP-dissociation inhibitors (GDIs)^40^,^41^ and microRNAs (miRNAs)^42^. Recent work has also highlighted the importance of interactions between the Rho GTPase signalling and the planar cell polarity pathway in controlling the assembly of apical actin filaments, as well as the docking and planar polarization of the basal bodies in MCCs^43^,^44^.

miRNAs or miRs are a class of small single-stranded and non-coding regulatory RNAs that control many biological processes by limiting the stability and the translation of their target mRNAs^45^,^46^. Abnormal miRNA activity has been associated with a wide variety of human pathologies including airway diseases^47^. We have previously demonstrated that the miR-34/449 family is important for the initiation of human and *Xenopus* MCC differentiation. Members of this family share high sequence homology and miR-449a/b/c, which are located on the same genomic locus as Multicilin, were identified as the most strongly induced miRNA species in human and *Xenopus* during MCC differentiation. We showed in these two species that miR-34/449 promote cell cycle exit and entry into differentiation by repressing several components of the cell cycle control machinery and of the Notch signalling pathway^9^. Their inactivation was sufficient to block centriole amplification and multiple motile cilia formation^9^. Two recent studies confirmed our findings by showing that miR-34/449-deficient mice exhibited impaired multiciliogenesis^48^,^49^. Song *et al*.^48^ also showed that the centriolar protein Cp110 is an additional important miR-34/449 target that must be repressed to allow the maturation of basal bodies. Thus, beyond their initial effect on cell cycle exit and entry into differentiation, miR-34/449 probably regulate later steps of the complex multiciliogenesis process. As the development of functional motile cilia appears exquisitely sensitive to the reorganization of the actin cytoskeleton, we reasoned that miR-34/449 may also regulate one or more molecules associated with actin dynamics or small GTPase pathways. Here we show that miR-34/449 indeed contribute to the establishment of the apical actin cytoskeleton, via a mechanism involving the direct repression of the small GTPase R-Ras. This further establishes miR-34/449 as a central control system of multiciliogenesis acting at several distinct levels of this complex physiological process.

## Results and Discussion

### The apical actin network of human and *Xenopus* MCCs

Apical actin cytoskeleton formation was examined at several time points during differentiation of primary cultures of human airway epithelial cells (HAECs) grown at an air–liquid interface (ALI) and in *Xenopus* embryonic epidermis^9^. Formation of the apical meshwork of filamentous actin (F-actin) was monitored directly by staining with fluorescent phalloidin and indirectly by staining for ezrin or phospho-ERM. In human and *Xenopus*, the acetylated tubulin-positive MCCs displayed a strong enrichment of apical F-actin (human: Supplementary Fig. 1a–d; *Xenopus*: Supplementary Fig. 1f). Basal bodies, which are positive for γ-tubulin labelling, are embedded within an apical F-actin and ezrin meshwork (Supplementary Fig. 1b). Reorganization of actin filaments involves cofilin, a ubiquitous G-actin-binding factor^50^. As unphosphorylated cofilin depolymerizes actin filaments, cofilin phosphorylation appears essential for cytoskeletal reorganization^50^. Phosphorylation of cofilin-1 also contributes to actin network stabilization and formation of focal adhesions^51^. Focal adhesion proteins indeed participate to ciliary adhesion complexes in MCCs and allow interactions between basal bodies and the apical actin network^33^. In HAECs, the levels of phosphorylated cofilin-1 and ezrin increased during MCC differentiation (Supplementary Fig. 1e). We noticed a parallel increase in the expression of the ERM-binding protein EBP50 (Supplementary Fig. 1e), an adapter protein required for the maintenance of active ERM proteins at the apical membrane of polarized epithelia^14^,^32^. Interestingly, we also observed a punctate labelling of phosphorylated cofilin-1, which was localized sub-apically close to basal bodies in MCCs, but was absent from non-ciliated cells (Supplementary Fig. 1c,d).

### miR-34/449 control apical actin network assembly in MCCs

**Figure 1:**
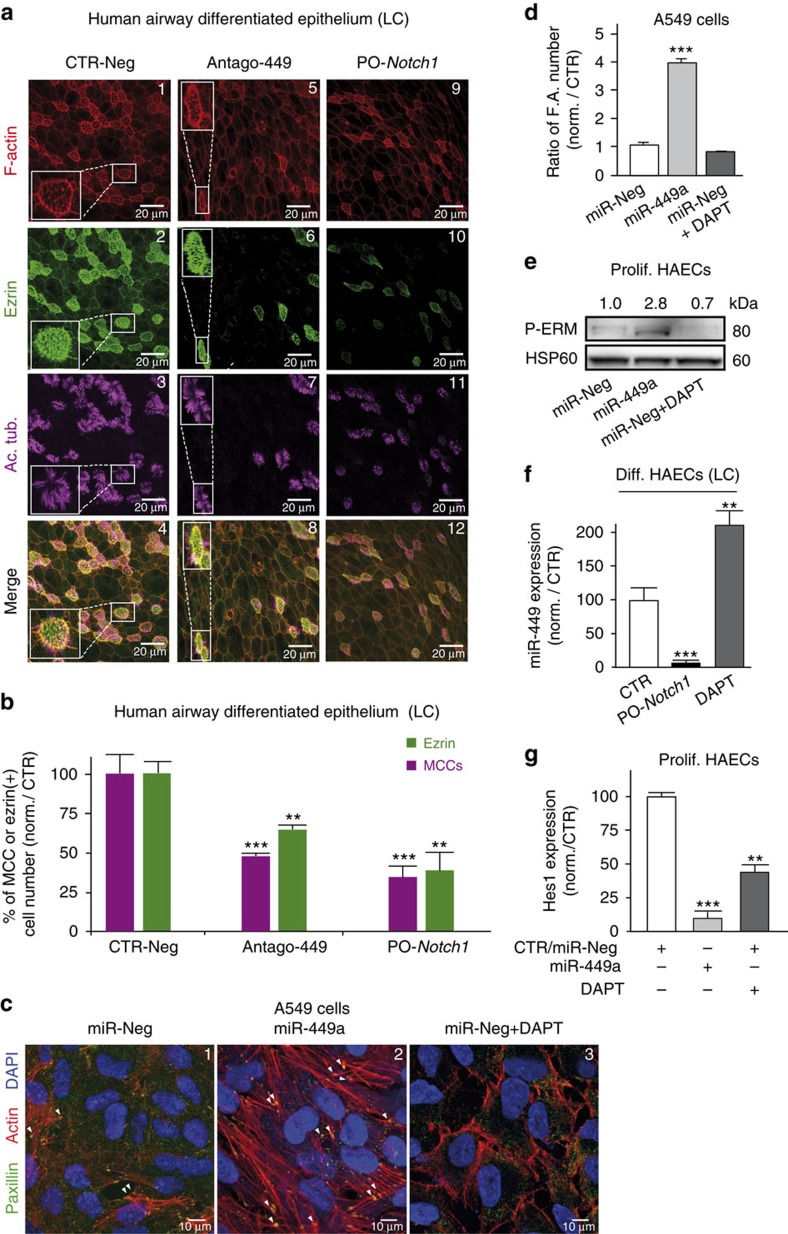
MiR-449 affects actin network remodelling and multiciliogenesis in HAECs. (**a**–**c**) Effect of a treatment by control antagomiR (CTR-Neg), anti-miR-449a/b (Antago-449) and miR-449::*Notch1* protector (PO-*Notch1*) on differentiating HAECs. (**a**) Staining for F-actin (**a**1,5,9), ezrin (**a**2,6,10) and acetylated tubulin (**a**3,7,11), at LC stage. (**b**) The histogram indicates the average percentage of MCCs (in magenta) and apical ezrin-positive (in green) cell number relative to control (means±s.d. from nine and three donors for MCC and ezrin quantifications, respectively. ***P<0.001, **P<0.01; Student's *t*-test). (**c**) Immunostaining of focal adhesions protein Paxillin (in green), F-actin (in red) and nuclei (in blue) in A549 epithelial cells transfected for 72 h with control miRNA (miR-Neg), miR-449a or miR-Neg plus 10 μM DAPT. (**d**) Ratio of focal adhesion number per cell, normalized to control (*n*=5 fields in three independent experiments; ***P<0.001; Student's *t*-test). (**e**) Effect of miR-449 overexpression and DAPT (10 μM) on ERM phosphorylation in proliferating HAECs. Phosphorylated protein levels were normalized with non-phosphorylated ERM and with an antibody against HSP60 as a loading control. Normalized fold changes are indicated on the corresponding bands. Experiments were representative of three donors. (**f**) Effect of PO-*Notch1* and DAPT (10 μM) in differentiating HAECs at LC stage on miR-449 expression, normalized with RNU44. (**g**) Real-time RT–PCR of *HES1* transcripts in control, DAPT (10 μM, 48 h)-treated or miR-449-overexpressing proliferating HAECs. Transcript levels of *HES1* were normalized against *UBC* transcript as an internal control. (**f**,**g**) Data represent the mean and s.d. of three independent experiments (***P<0.001, **P<0.01; Student's *t*-test).

**Figure 2:**
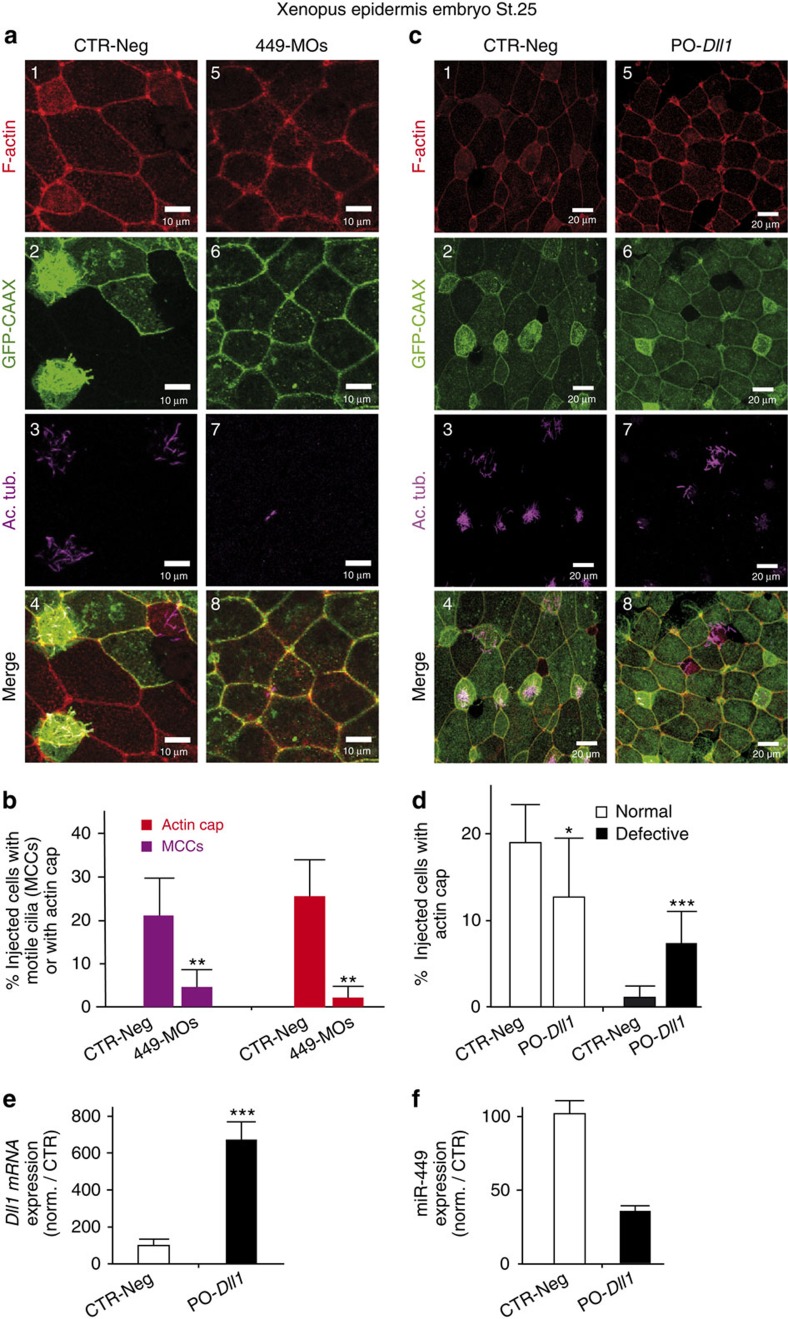
MiR-449 affects actin network remodelling and multiciliogenesis in *Xenopus* epidermis. (**a**–**d**) The epidermis precursor blastomeres (eight-cell stage *Xenopus* embryos) were injected with negative control morpholinos (CTR-Neg) (**a**,**c**), with morpholinos against miR-449 (449-MOs) (**a**,**b**) or with protector morpholinos of *Dll1* (PO-*Dll1*) (**c**,**d**). Staining at stage 25 for F-Actin (in red, **a**1,5 and **c**1,5) and motile cilia (Ac. Tub. in magenta, **a**3,7 and **c**3,7). Injected cells were detected by the expression of a synthetic mRNA coding for membrane-bound GFP (GFP-CAAX in green, **a**2,6 and **c**2,6). (**b**) The histogram indicates the percentage of GFP-CAAX-positive injected cells that develop motile cilia or apical actin cap in controls (Stage 24+25: *n*=5 fields/583 injected cells) and in miR-449 morphants (Stage 24+25: *n*=8 fields/625 injected cells; *P*-value _st.24+25_=0.0087; Mann–Whitney test with two-tailed *P*-value). (**d**) Percentage of injected cells positive for GFP fluorescence with normal or defective actin cap in control (*n*=30 fields per 1,345 injected cells) versus PO-*Dll1* morphants (*n*=32 fields per 1,268 injected cells; *P*-value (normal versus defective in control) _st.24+25_ <0.0001, *P* value (normal versus defective in PO-*Dll1*-injected _st.24+25_=0.0033; Mann–Whitney test with two-tailed *P*-value). (**e**) Effect of protecting the *Dll1* mRNA from the interaction with miR-449 (PO-*Dll1*) in *Xenopus* epidermis on *Dll1* expression (normalized with *ornithine decarboxylase* (ODC)). (**f**) Effect of PO-*Dll1* in *Xenopus* epidermis on miR-449 expression, normalized with U6. Data are means±s.d. of two independent experiments.

We examined whether the miR-34/449 family can control the formation of the apical actin network, a prerequisite for basal body anchoring and cilium elongation. To invalidate miR-449 or miR-34b/c activity in human, we transfected HAECs with a cholesterol-conjugated antagomiR directed against miR-449 (Antago-449) or against miR-34 (Antago-34) and assessed MCC differentiation. Antago-449 as well as antago-34 strongly blocked miR-449a/b, whereas antago-34 blocked miR-34a/b/c more efficiently than antago-449 (Supplementary Fig. 2b). Antago-449 and antago-34 blocked to the same extent the formation of MCCs and no additive effect was observed (Supplementary Fig. 2c). The huge induction of miR-449 expression at early ciliogenesis (stage EC) suggests that early effects were mainly mediated through miR-449, without excluding a later role for miR-34 (Supplementary Fig. 2a). This could also explain the lack of compensation of miR-34 in our antago-449 conditions, which do not alter the expression of miR-34. In HAECs, miR-449 silencing caused a decrease of 51%±3.5 in the number of acetylated tubulin-positive cells and of 33%±7 in the number of ezrin-positive cells (Fig. 1b). In *Xenopus* epidermis, miR-449a and miR-34b were both specifically expressed in MCCs, and their levels in the developing epidermis showed a similar evolution (Supplementary Fig. 2d, see also ref. 9). Previously, we knocked down miR-449 in *Xenopus* MCCs by injecting a cocktail of morpholino antisense oligonucleotides against miR-449a/b/c (449-MOs) into the prospective epidermis at the eight-cell stage^9^. In *Xenopus*, miR-34b was detected in MCCs (Supplementary Fig. 2d–f), and *in situ* hybridization (ISH) experiments also revealed that 449-MOs not only blocked the expression of miR-449 but also blocked the expression of miR-34b (Supplementary Fig. 2f), suggesting that 449-MOs collectively inhibit miR-34/449 miRNAs (Supplementary Fig. 2e,f). MiR-449 knockdown suppressed multiciliogenesis and apical actin web formation in both HAECs (Fig. 1a,b) and *Xenopus* embryonic epidermis (Fig. 2a,b). In embryos injected with 449-MOs, the number of acetylated tubulin-positive cells went down to 18%±13 of the control and the number of apical actin cap-positive cells decreased to 9%± 8 of the control (Fig. 2a/b). These results establish that the miR-34/449 family interferes with MCC apical actin meshwork formation in both models. As miR-34 and miR-449 miRNAs share the same targets, we only used miR-449 in the rest of this study. The impact of miR-449 on the actin cytoskeleton was further investigated by monitoring the formation of focal adhesion and stress fibres, which are thick and relatively stable actin filaments involved in cell adhesion and morphogenesis^52^,^53^. We found that in proliferating A549 cells, a human lung cell line devoid of miR-449 and miR-34b/c, miR-449 overexpression increased actin stress fibres and focal adhesion formation (Fig. 1c,d, see also Supplementary Fig. 3d,e). In addition, western blot analysis revealed increased ERM phosphorylation following miR-449 transfection in proliferating HAECs (Fig. 1e), consistent with the regulatory role of phospho-ERM during actin cytoskeleton dynamics^50^,^54^. Thus, our data show that the miR-34/449 family clearly contributes to actin cytoskeleton remodelling in several independent models. Next, we addressed the precise mode of action of miR-34/449 in the construction of the apical actin network in MCCs.

### Mutual repression between miR-449 and the Notch pathway

As the miR-34/449 family represses the Notch pathway during MCC differentiation^9^, we assessed the contribution of the Notch signal to the actin web reorganization. We treated proliferating HAECs or human lung A549 cells (which both are devoid of endogenous miR-449) either with miR-449 or with N-[N-(3,5-Difluorophenacetyl)-L-alanyl]-S-phenylglycine t-butyl ester (DAPT), a γ-secretase inhibitor that blocks Notch activation. As expected, both ectopic expression of miR-449 in proliferating HAECs and DAPT repressed the expression of the Notch target gene *HES1* (Fig. 1g). In proliferating A549 cells, Notch inhibition with DAPT alone had neither impact on the formation of actin stress fibres and focal adhesions (Fig. 1c,d) nor on ERM phosphorylation in proliferating HAECs (Fig. 1e). This suggests that miR-449 cause a rise of levels of phosphorylated ERM independently of Notch repression.

We observed that preventing the binding of miR-34/449 on *Notch1* (PO-*Notch1* in HAECs, Fig. 1a,b) or on the Notch ligand *Dll1* (PO-*Dll1* in *Xenopus*, Fig. 2c–f) with protector oligonucleotides coordinately blocked multiciliogenesis and apical actin network formation. This is consistent with the need for an early repression of the Notch pathway by miR-34/449, to allow MCC differentiation. Of note, in *Xenopus*, the percentage of cells exhibiting defective apical actin meshwork was higher in miR-34/449 morphants (Fig. 2b) than in PO-*Dll1* morphants (Fig. 2d), suggesting that miR-34/449 may affect additional targets. We also noticed a reduction of miR-449 levels when preventing miR-34/449 binding on *Notch1* in differentiating HAECs (Fig. 1f) and on *Dll1* in frog epidermis (Fig. 2f). Conversely, miR-449 expression was increased after treatment of differentiated HAECs with DAPT (Fig. 1f). Altogether, these data reveal the existence of a double-negative feedback loop between miR-449 and the Notch pathway. We hypothesize that once this loop is locked in a state of high miR-449 expression, interactions of miR-449 with additional targets expressed at subsequent steps of multiciliogenesis remain possible (see Fig. 8d). According to a recent work, CP110 would represent one such target. Its repression by miR-34/449 appears to affect centriole maturation, but not apical actin network assembly^48^. We thus looked for other possible targets of miR-34/449 that would be directly related to actin dynamics.

### miR-34/449 targets components of the small GTPase pathways

**Figure 3:**
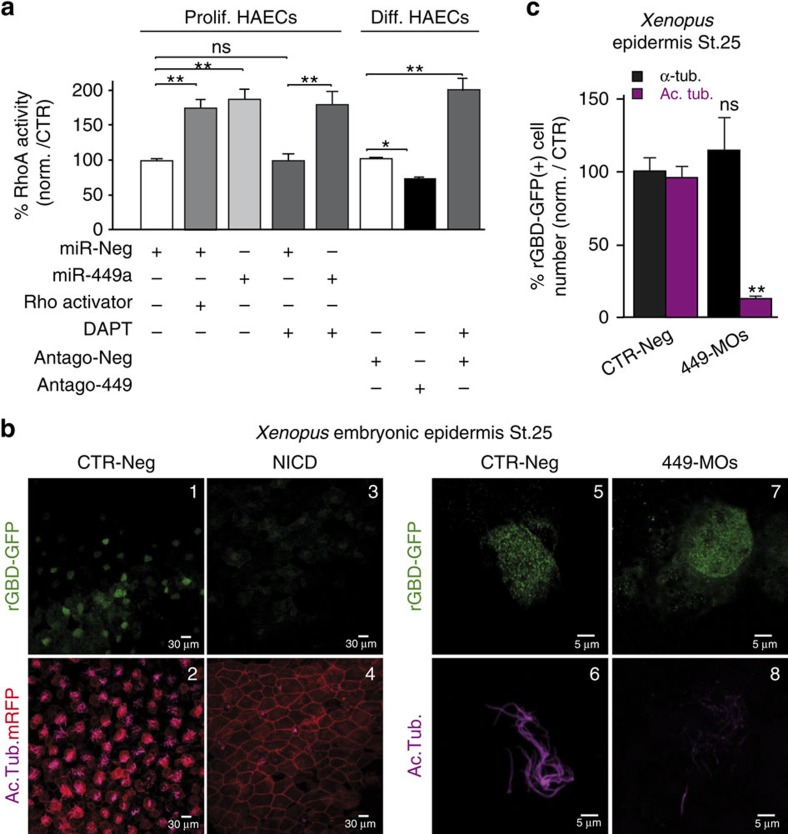
miR-449 controls small GTPase pathways during MCC differentiation. (**a**) Left (Prolif. HAECs): RhoA activity in proliferating HAECs transfected for 72 h with miR-Neg, miR-449a and/or incubated with DAPT (10 μM) or a Rho activator (calpeptin, 1 U ml^−1^, 2 h). Right (Diff. HAECs): RhoA activity in differentiating HAECs at Po stage treated for 72 h with antago-Neg, antago-449 or DAPT (10 μM). RhoA activity is expressed as a percentage relative to control. Data are mean±s.d. from at least three independent experiments (*P<0.05, **P<0.01 and ***P<0.001; Student's *t*-test). (**b**) RhoA activity in *Xenopus* epidermis at stage 25, assessed by measuring the fluorescence of rhotekin rGBD-GFP, an active RhoA sensor (green, upper panels). Injected cells are identified by the membrane-bound RFP (mRFP in red, lower panels). Cells were either untreated (CTR-Neg, **b**1,2,5,6), injected with miR-449 morpholinos (449-MOs, **b**7,8) or with Notch intracellular domain NICD (b3–4). Motile cilia's staining is in magenta (lower panels). (**c**) The histogram indicates the percentage of rGBD-GFP-positive cells stained for α-tubulin mRNA (α-Tub. in black) or acetylated tubulin (Ac. Tub. in magenta) in miR-449 morphants (449-MOs, *n*=68) relative to negative control (*n*=110). Data are mean±s.e.m. (**P<0.005, ns, not significant, one-way analysis of variance with Dunnett's test).

Several small GTPase proteins such as RhoA or Rac1 act as key regulators of multiciliogenesis^14^,^27^,^55^,^56^. We assessed the functional impact of miR-449 on the activity of RhoA and Rac1,2,3 by transfecting proliferating HAECs with miR-449 and differentiated HAECs with antago-449. In proliferating primary HAECs, miR-449 overexpression caused a 50% increase in the level of active RhoA-GTP, similar to the effect of the Rho activator calpeptin (Fig. 3a), while it decreased by 35±14% Rac1,2,3 activity (Supplementary Fig. 3a), as previously observed in another cellular context with miR-34a (ref. 57). In contrast, Notch pathway inhibition by DAPT had no impact on RhoA activity in proliferating HAECs (Fig. 3a), indicating that exogenous miR-449 modulated the RhoA pathway in a Notch-independent manner in this assay. MiR-449 silencing caused a modest but significant reduction of RhoA activity in differentiating HAECs (Fig. 3a). Conversely, DAPT caused a significant increase in RhoA activity in differentiating HAECs (Fig. 3a), consistent with the concomitant upregulation of miR-449 expression (Fig. 1f)^9^. RhoA activation was also examined in *Xenopus* MCCs by injecting embryos with an RNA encoding the Rhotekin rGBD-GFP, a sensor of activated RhoA^56^. The rGBD-GFP signal was detected in MCCs from control embryos but not in embryos injected with Notch-ICD, in which MCC differentiation was abolished (Fig. 3b). In control embryos and in miR-34/449 morphants, almost all rGBD-GFP-positive cells expressed the early MCC differentiation marker α-tubulin (Fig. 3c). By contrast, in miR-34/449 morphants only 13% of rGBD-GFP-positive cells displayed acetylated tubulin ciliary staining, compared with 96% in the control situation (Fig. 3b,c). Thus, following the experimental inhibition of miR-34/449 in *Xenopus*, RhoA activation can still be detected in MCCs unable to grow cilia. However, we cannot rule out discrete changes in the sub-cellular localization of activated RhoA in miR-34/449-deficient embryos.

Collectively, these results suggest that, although the modulation of RhoA activity by miR-34/449 may play a role in the control of apical actin polymerization, other miR-34/449 targets contribute to the profound disruption of the actin cap observed after miR-34/449 inactivation.

In a bid to identify such additional factors, we applied several miRNA target prediction tools^58^ to identify putative miR-34/449 targets among the small GTPase pathways. Specifically, we looked for relevant miR-34/449 mRNA targets that were repressed during MCC differentiation and after overexpression of miR-34/449 in proliferating HAECs (Gene Expression Omnibus (GEO) data set GSE22147). This survey led us to identify several candidates related to small GTPase signalling and actin cytoskeleton remodelling: (1) ARHGAP1, a member of the Rho-GAP family^59^; (2) ARHGDIB, also called Rho-GDI2 (ref. 40); (3) DAAM1, the diaphanous-related formin Dishevelled-associated activator of morphogenesis^60^; (4) NDRG1, N-myc downstreamregulated gene 1, an iron-regulated metastasis suppressor^61^; (5) R-Ras, a member of the superfamily of small GTPases, related to Ras^38^.

By looking more precisely at the three major families of regulators of small GTPases expressed during HAECs differentiation or after miR-34/449 transfection, we detected 23 distinct ARHGAPs, including *ARHGAP1*; we also detected 22 distinct ARHGEF transcripts but none of them were predicted as direct miR-34/449 targets; regarding the three known mammalian *ARHGDI* transcripts^40^, only *ARHGDIB* was further analysed, as *ARHGDIA* expression levels did not change during HAECs differentiation or after miR-34/449 transfection and *ARHGDIG* was not detected (Supplementary Fig. 3b, see also GEO GSE22147).

**Figure 4:**
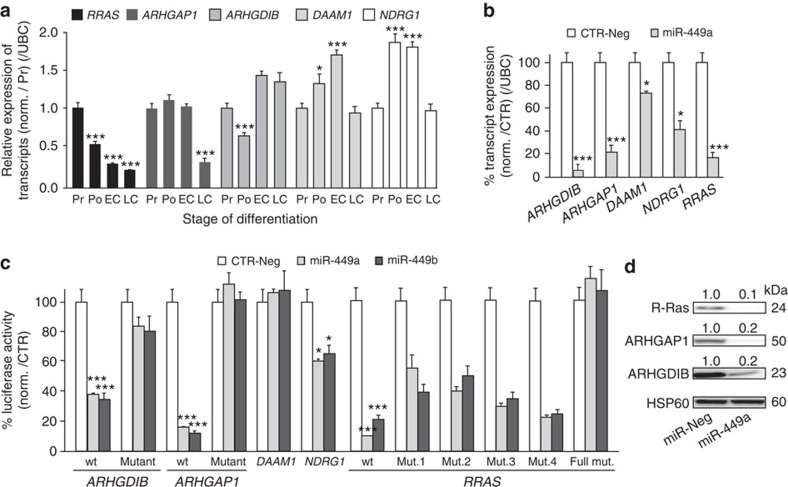
*ARHGAP1*, *ARHGDIB* and *RRAS* are targeted by miR-449 in HAECs. (**a**,**b**) Transcript expression levels of *ARHGAP1*, *ARHGDIB*, *DAAM1*, *NDRG1* or *RRAS* were analysed using real-time RT–PCR during HAEC differentiation (**a**) or following miR-449a overexpression (48 h) in proliferating HAECs (**b**), and normalized with *UBC* transcript as an internal control. (**c**) Specific interaction between miR-449a/b and the 3′-UTRs of *ARHGAP1*, *ARHGDIB*, *DAAM1*, *NDRG1* and *RRAS* mRNAs was assessed using luciferase reporter assay on constructs carrying either the wild-type (wt) or mutants (mut.) 3′-UTR-binding sites for miR-449. Values were normalized with the internal *Renilla* luciferase control. (**d**) Detection of ARHGAP1, ARHGDIB and R-Ras proteins after miR-449 overexpression in proliferating HAECs for 72 h. HSP60 is used as an internal control. Quantification of protein levels are indicated above each corresponding band. All data are means±s.d. of at least three independent experiments (***P<0.001, **P<0.01 and *P<0.05; Student's *t*-test).

**Figure 5:**
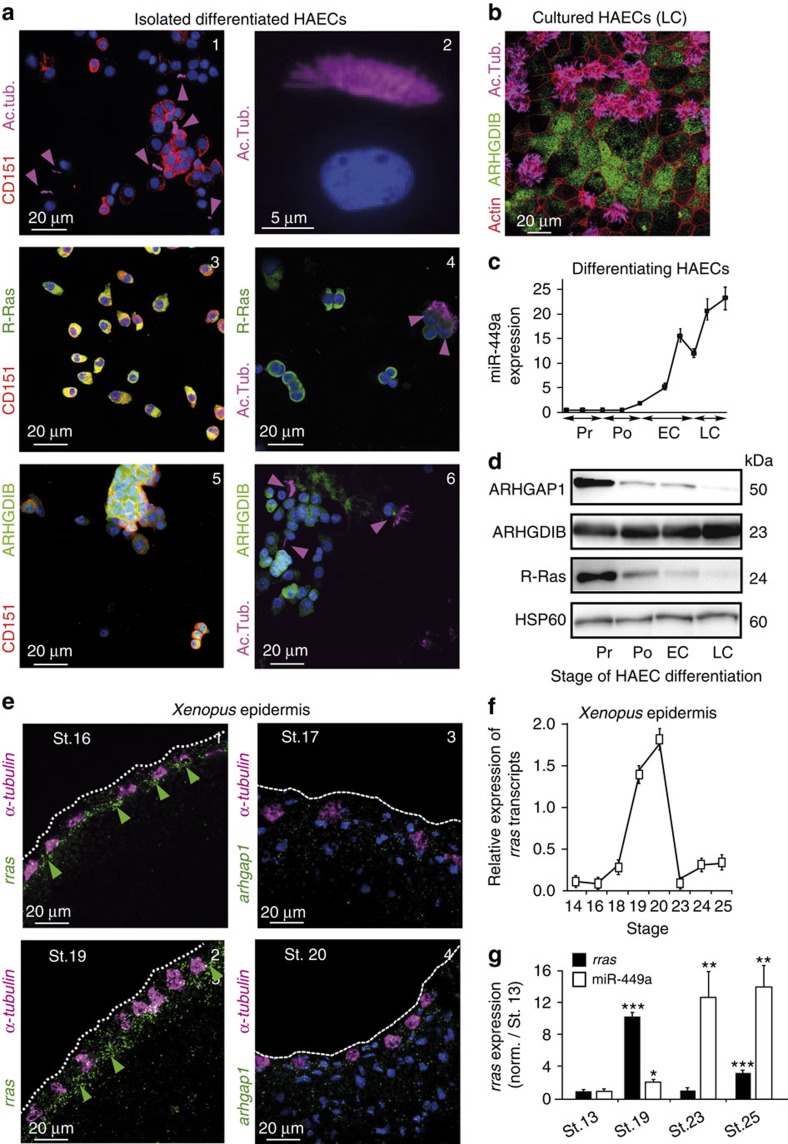
Cellular specificity of expression of ARHGAP1, ARHGDIB and R-Ras during MCC differentiation. (**a**,**b**) Fluorescence immunocytochemistry experiments on cytospins from dissociated HAECs (**a**1–6) and primary HAEC cultures at LC step (**b**) illustrate the cell-specific expression of human ARHGDIB (in green, **a**5–6,**b**) and R-Ras proteins (in green, **a**3,4). Basal and ciliated cells are CD151+ (in red, a1,3,5) or acetylated tubulin+ (in magenta a2,4,6 and **b**), respectively. Panel **a**2 is a magnification of an isolated acetylated tubulin-positive MCC. HAECs are stained for nuclei with 4,6-diamidino-2-phenylindole (DAPI; in blue, **a**1–6), F-Actin with phalloidin (in red, **b**). Expression levels of miR-449a (**c**) and of ARHGAP1, ARHGDIB and R-Ras proteins (**d**) during HAEC differentiation. MiR-449 levels are normalized with *RNU44* (**c**) and HSP60 was used as a loading control (**d**). (**e**) FISH analysis of *rras* (**e**1,2) and *arhgap1* (**e**3,4) mRNA on sections of *Xenopus* embryonic epidermis at stages 16 and 19 (**e**1,2) or at stages 17 and 20 (**e**3,4). MCC precursors are positive for *α-tubulin* (magenta; **e**1–4). DAPI staining is in blue and white dotted lines indicate the surface of the outer layer. (**f**) Real-time RT–PCR of *rras* transcripts in *Xenopus* epidermis performed at each corresponding stages indicted in the figure. (**g**) Transcript levels of *rras* decrease concomitantly with the induction of miR-449 expression. Transcript levels of *rras* and miR-449 were normalized and compared with their respective values obtained in stage 13. Transcript levels of *rras* were normalized against *Odc* transcript as an internal control and miR-449 expression was normalized with U2 as an internal control. Data represent the mean and s.d. of three independent experiments (***P<0.001, **P<0.01 and *P<0.05; Student's *t*-test).

The transcripts *ARHGAP1*, *ARHGDIB*, *DAAM1*, *NDRG1* and *RRAS* are all modulated during HAEC differentiation (Fig. 4a, see also GEO GSE22147). Figure 4a shows that the transcript level of *ARHGAP1*, *DAAM1* and *NDRG1* decreased at late ciliogenesis (LC). The expression of *ARHGDIB* transcript slightly decreased at the onset of differentiation (that is, polarization step, Po) but rose again during the phase of multiciliogenesis (Fig. 4a). The *RRAS* transcript level decreased throughout the whole time course of MCC differentiation (Fig. 4a). In proliferating HAECs, miR-449 overexpression strongly reduced the transcript levels of *ARHGDIB*, *ARHGAP1* and *RRAS*, whereas the expression of DAAM1 and *NDRG1* transcripts was slightly decreased (Fig. 4b). These putative targets were further investigated using a dual luciferase reporter assay in HEK293 cells. MiR-449a and miR-449b reduced the relative luciferase activity of chimeric constructs containing the wild-type 3′-untranslated regions (3′-UTRs) of *ARHGAP1*, *ARHGDIB*, *NDRG1* and *RRAS*, but not of *DAAM1* 3′-UTR (Fig. 4c). Next, we focused on the three most significantly regulated miR-34/449 targets *ARHGAP1*, *ARHGDIB* and *RRAS*. MiR-449-mediated silencing of either *ARHGAP1*, *ARHGDIB* or *RRAS* was respectively abolished when miR-34/449-predicted binding sites were mutated (Fig. 4c). Among the four miR-449-binding sites in the 3′-UTR of *RRAS*, the strongest effect was observed for the most 3′-site, which also corresponds to the unique conserved site between human and *Xenopus* (Fig. 4c and Supplementary Fig. 4a). The protein levels of ARHGAP1, ARHGDIB and R-Ras proteins were also dramatically decreased after transfection of proliferating HAECs with miR-449 (Fig. 4d). These results establish *ARHGAP1*, *ARHGDIB* and *RRAS* transcripts as *bona fide* targets of miR-34/449. Consistent with this conclusion, both ARHGDIB and R-Ras proteins were excluded from acetylated tubulin-positive MCCs, while being enriched in non-ciliated CD151-positive basal HAECs^9^,^62^ (Fig. 5a,b). This is consistent with a recent gene expression profiling study performed in mouse trachea, which reported a higher level of expression of *RRAS* transcripts in non-ciliated cells than in ciliated cells^63^ (GSE42500). We could not address this possibility for ARHGAP1 as none of the antibodies that we tested worked in immunofluorescence. In differentiating HAECs, both R-Ras and ARHGAP1 protein level strongly and continuously decreased from Po to LC, concomitantly with the increase in miR-449 expression (Fig. 5c,d). Conversely, ARHGDIB protein level increased during human HAEC differentiation (Fig. 5d). As ARHGDIB protein was mainly excluded from human MCCs (Fig. 5a,b), the global protein increase observed during HAEC differentiation in western blotting is probably explained by its stronger expression in non-ciliated HAECs. In *Xenopus* epidermis, *rras* expression measured by quantitative reverse transcriptase–PCR (qRT–PCR) became detectable at neurula stage 16, then dramatically increased until stage 20, before multiciliogenesis and subsequently dropped (Fig. 5f,g). Interestingly, *rras* transcript levels were anti-correlated with miR-449a levels during the course of MCC differentiation (Fig. 5g). When analysed by ISH, *rras* transcripts were primarily detected in inner-layer cells that were negative for the MCC marker *α-tubulin*, at stages 16 and 19 (Fig. 5e). In contrast, *arhgap1* expression was very faint at similar stages and did not show any specific pattern of distribution (Fig. 5e). No *arhgdib* signal was detected in the *Xenopus* epidermis at any developmental stage (Supplementary Fig. 3i).

The silencing of *ARHGDIB*, *ARHGAP1* and *RRAS* using specific small interfering RNAs (siRNAs) in proliferating HAECs strongly reduced the level of expression of the corresponding proteins (Supplementary Fig. 3c). In proliferating human A549 cells, the silencing of *RRAS* or *ARHGAP1* but not *ARHGDIB* increased actin stress fibres and focal adhesion formation (Supplementary Fig. 3d,e), and mimicked the effects observed after miR-449 overexpression (Fig. 1c,d and Supplementary Fig. 3d,e). These effects were abolished when cells were treated with an inhibitor of Rock (Y27632), an important RhoA effector^55^ (Supplementary Fig. 3e). In proliferating HAECs, we also found that RhoA activity increased after silencing of *RRAS* but not of *ARHGAP1* or *ARHGDIB*. This effect remained however smaller than the one induced by miR-449 expression (Supplementary Fig. 3f). Rac1,2,3 activity was slightly reduced by miR-449 expression, whereas it was not affected by a silencing of *RRAS* (Supplementary Fig. 3a). These observations point to the participation of miR-34/449 and R-Ras to actin network reorganization and their capacity to alter the RhoA activity. They are in agreement with a previous work showing that *RRAS* deficiency is associated with cortical actin reorganization in adult haematopoietic progenitor cells^38^. Our data suggest that the induction of RhoA activity by miR-449 may at least in part involve the silencing of *RRAS*, notwithstanding possible contributions by additional regulators. The lack of effect on RhoA activity after *ARHGAP1* and *ARHGDIB* silencing is probably in line with the existence of redundant or compensatory mechanisms controlling RhoA activity in the context of multiciliogenesis. As the pattern of expression of both *arhgap1* and *arhgdib* in *Xenopus* epidermis was not consistent with an association to MCC precursors, *arhgap1* and *arhgdib* were not further analysed in *Xenopus*.

We designed target protection assays in which cholesterol-conjugated modified oligonucleotides were transfected in differentiating HAECs to compete with the binding of miR-34/449 on the site identified within the human 3′-UTR of *ARHGDIB* mRNA (PO-*ARHGDIB*). No effect was observed either on apical actin web or multiciliogenesis after protection with PO-*ARHGDIB* or after *ARHGDIB* silencing with siRNAs in differentiating HAECs (Supplementary Fig. 3g,h). Incidentally, the existence of five miR-34/449-binding sites in the 3′-UTR of *ARHGAP1* made elusive the assessment of a target protection of *ARHGAP1* against a miR-34/449 action in primary HAEC cultures. Intriguingly, silencing *ARHGAP1* in human at an early step of HAEC differentiation strongly affected apical actin meshwork formation and blocked multiciliogenesis (Supplementary Fig. 3g,h), suggesting that *ARHGAP1* does play a role in multiciliogenesis, in which its level of expression has to be finely controlled during maturation of MCCs.

These data posit the absence of R-Ras in miR-34/449-expressing MCCs as a conserved feature across tetrapods. We therefore focused on the functional impact of miR-449-mediated repression of *RRAS* on apical actin reorganization in MCCs.

### miR-34/449 control apical actin assembly by repressing R-Ras

We used target protection assays (cholesterol-conjugated modified oligonucleotides in HAECs or morpholino oligonucleotides in frog epidermis) to compete with the binding of miR-34/449 on sites identified within the human and *Xenopus* 3′-UTRs of *RRAS* mRNA. In human cells, the *RRAS* protector oligonucleotide (PO-*RRAS*) strategy effectively blocked the action of ectopic miR-449, as evidenced on *RRAS* 3′-UTR in luciferase assays (Supplementary Fig. 4b). In addition, miR-34/449 knockdown or protection of *RRAS* mRNA from miR-34/449 in frog epidermis led to an increase in *rras* transcript levels (Supplementary Fig. 4c). We checked that in both species the expression of *RRAS2*, an *RRAS*-related gene, did not interfere with R-Ras signalling. *RRAS2* expression remained at a very low level during MCC differentiation and in response to miR-449 overexpression, and was not altered by miR-34/449 knockdown or PO-*RRAS* protection (HAECs, see GEO GSE22147; *Xenopus*, Supplementary Fig. 4c). In both models, the PO-*RRAS* strategy was also able to increase endogenous R-Ras protein level (Supplementary Fig. 4d,e). Collectively, these assays unambiguously establish that *RRAS* transcripts were specifically targeted by miR-34/449 in human and *Xenopus* MCCs.

**Figure 6:**
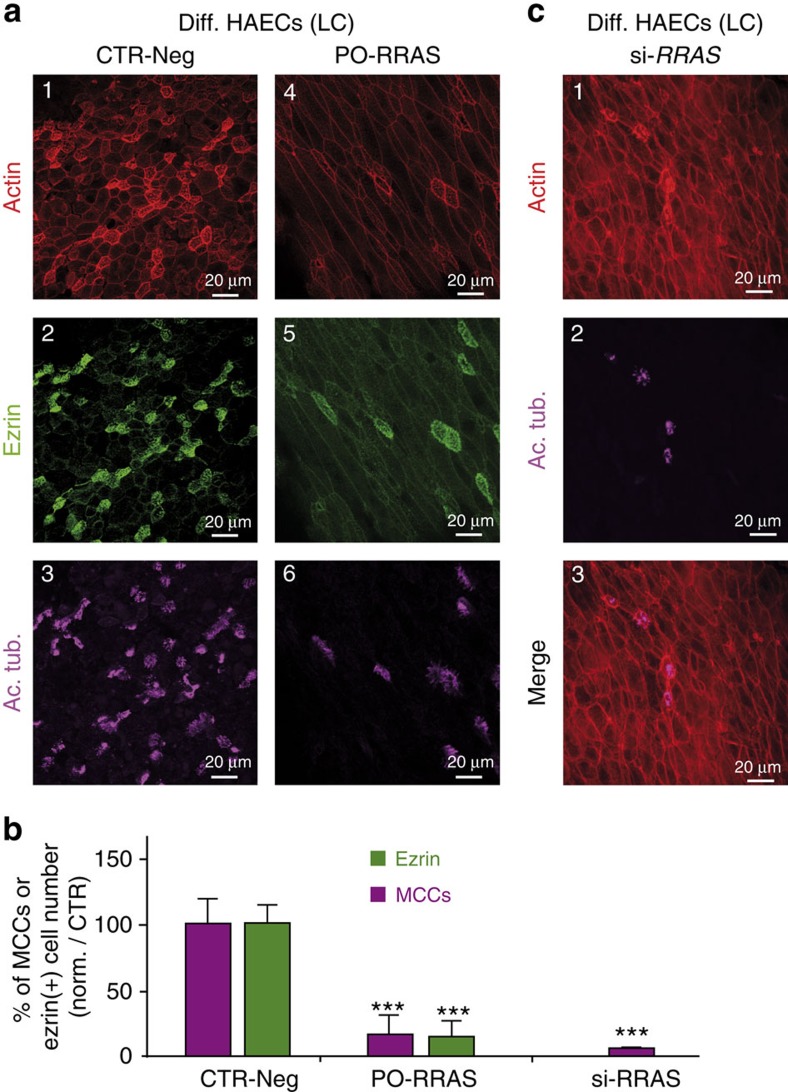
The direct repression of *RRAS* by miR-449 affects multiciliogenesis and apical actin cytoskeleton reorganization in HAECs. (**a**) Differentiating HAECs were chronically treated with negative oligonucleotide (CTR-Neg, a1–3) or with an oligonucleotide protecting the miR-449-binding site on *RRAS* (PO-*RRAS*, **a**4–6) and stained at LC stage for F-actin (**a**1,4), ezrin (**a**2,5) and acetylated tubulin (Ac. Tub., **a**3,6). (**b**) Alternatively, differentiating HAECs were transfected at seeding time with si-*RRAS* and stained at LC stage for F-actin (in red, **c**1) and motile cilia (Ac. Tub. in magenta, **c**2). (**c**) The histogram indicates the number of apical ezrin-positive cells (in green) and MCCs (in magenta) normalized in percentage from control. Protecting the *RRAS* mRNA from interaction with miR-449 leads to defects in apical actin reorganization together with a decrease in MCC differentiation similar to that observed after inhibition of miR-449 activity. All data are means±s.d. from at least three independent experiments (***P<0.001; Student's *t*-test).

**Figure 7:**
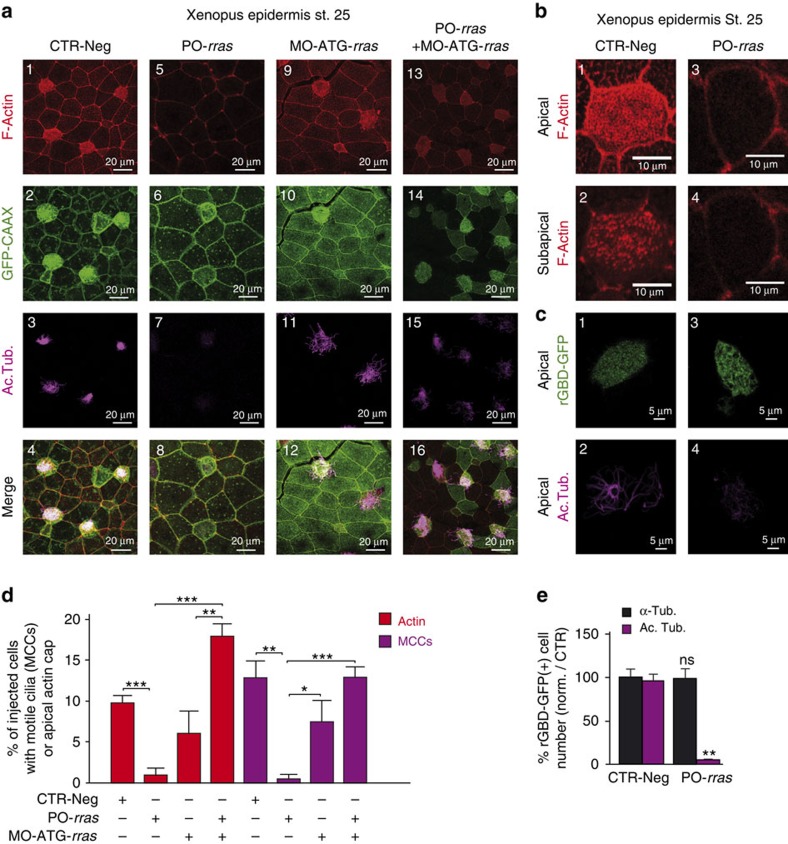
The direct repression of *RRAS* by miR-449 affects multiciliogenesis and apical actin cap formation in *Xenopus*. (**a**,**b**) Eight-cell stage *Xenopus* embryos were injected in the epidermis precursor blastomeres with a mixture of synthetic mRNA coding for GFP-CAAX to label the injected cells (in green, **a**2,6,10,14) and negative control MO (CTR-Neg, **a**1–4, **b**1,2), or a morpholino protecting *rras* against binding by miR-449 (PO-*rras*, **a**5–8, **b**3,4) or a morpholino blocking the translation of *rras* (MO-ATG-*rras*, **a**9–12), or a combination of PO-*rras* and MO-ATG-*rras* (**a**13–16). MCCs are stained with an anti-acetylated tubulin antibody (in magenta, **a**3,7,11,15 and **c**2,4), F-actin with phalloidin (red, **a**1,5,9,13 and **b**1–4). (**b**) The impact of Po-*rras* in MCCs on apical (upper **b**5) and sub-apical actin (lower **b**6) was observed with F-actin staining. (**c**) In independent experiments, we used rGBD-GFP to examine RhoA activity in *Xenopus* MCCs (**c**1–4). Eight-cell stage *Xenopus* embryos were injected in the epidermis precursor blastomeres with a reporter of RhoA activity (rGBD-GFP in green, **c**1–4) and with negative control MO (CTR-Neg, c1–2) or with PO-*rras* (c3–4). MCCs are stained in magenta (**c**2,4). Protecting the *rras* mRNA from interaction with miR-449 results in defects in actin cap formation (F-Actin, **a**5 and **b**3,4) together with a loss of MCCs (Ac. Tub., **a**7,**c**4) without affecting apical RhoA activity (rGBD-GFP, **c**1,3). This phenotype is rescued when the translation of the protected *rras* mRNA is blocked by coinjection of MO-ATG-*rras* (**a**13–16). (**d**) The histogram indicates the percentage of injected cells (positive for mGFP) that develop proper apical actin cap (in red) and motile cilia (in magenta) in *Xenopus* epidermis at stage 25. CTR-Neg, *n*=10 embryos per 413 injected cells; PO-*rras*, *n*=8 embryos per 350 injected cells; MO-ATG-*rras*, *n*=8 embryos per 290 injected cells; MO-PO-*rras*+MO-ATG-*rras n*=9 embryos per 395 injected cells (***P=0.009 and *P*<0.0001, and **P=0.0016; Mann–Whitney test). Data are mean±s.e.m. (**e**) The histogram indicates the percentage of rGBD-GFP-positive cells stained for α-tubulin (α-tub. in black) or acetylated tubulin (Ac. Tub. in magenta) in PO-*rras* morphants (PO-*rras*, *n*=150) in comparison with the negative control (CTR-Neg, *n*=110). (**P<0.005; ns, no significant; one-way-analysis of variance with Dunnett's test). Data are mean±s.e.m.

In human (Fig. 6a,b) and in *Xenopus* (Fig. 7a,d), protection of the *RRAS* transcript from miR-34/449 binding led to a strong reduction in apical actin meshwork and motile cilia formation. Silencing *RRAS* in human at an early step of HAEC differentiation also strongly affected apical actin meshwork formation and blocked multiciliogenesis (Fig. 6b,c). These results strongly suggest that R-Ras plays a key role in maturation of MCCs, in which its level of expression has to be tightly fine-tuned, to allow apical actin cap formation and multiciliogenesis. In frog epidermis, both apical and sub-apical phalloidin staining were altered in PO-*rras*-injected embryos (Fig. 7a,b). In contrast, F-actin staining remained detectable at cellular junctions in both models. Importantly, actin cap formation and multiciliogenesis were rescued in *Xenopus* epidermis when a morpholino designed to block R-Ras translation (MO-ATG-*rras*) was co-injected with PO-*rras* (Fig. 7a,d). At the dose used for this assay, injection of MO-ATG-*rras* alone had no significant effect on either apical actin meshwork formation or multiciliogenesis (Fig. 7a–d), suggesting that *rras* expression may be already repressed by the presence of endogenous miR-34/449 in those MO-ATG-*rras* maturing MCCs.

Our data unambiguously indicate that the repression of *RRAS* at a late step of MCC differentiation by miR-34/449 is required for apical actin network assembly and multiciliogenesis in human (Fig. 6) as well as in frog (Fig. 7). Considering that R-Ras activity can be increased by Notch pathway activation, as previously observed in another cellular model^39^, and considering the early repression of Notch signalling by miR-34/449 during vertebrate multiciliogenesis^9^, miR-34/449 is therefore able to control R-Ras function at two distinct levels: (1) directly, via the inhibition of *RRAS* expression, and (2) indirectly, via the inhibition of R-Ras activity through the repression of the Notch pathway.

**Figure 8:**
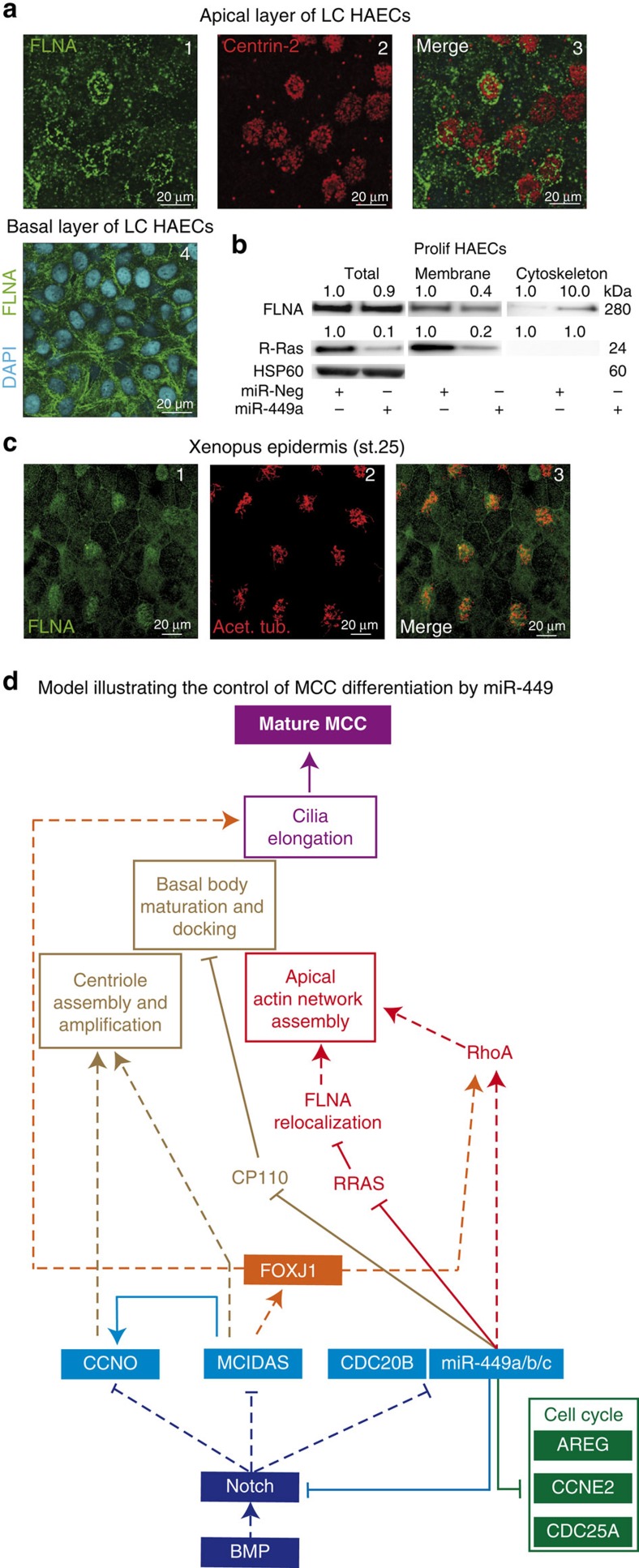
MiR-34/449 promote FLNA relocalization during MCC differentiation. (**a**) Apical layer of differentiated HAECs (LC stage) were stained for the basal bodies marker centrin-2 (in red) and the actin-binding protein filamin-A (FLNA, in green). FLNA labelling was enriched in the apical layer near basal bodies (**a**1–3). Basal layer of differentiated HAECs (LC stage) were stained for FLNA (in green) and for nuclei with 4,6-diamidino-2-phenylindole (DAPI; in blue) (**a**4). (**b**) Modulation of protein levels of FLNA and R-Ras in total fraction (Total), membrane fraction (Membrane) and cytoskeletal fraction (Cytoskeleton) isolated from proliferating HAECs transfected for 72 h with miR-Neg or miR-449a as indicated each below corresponding band. Protein levels were normalized against HSP60 as an internal control for the total fraction and normalized fold changes are indicated above the corresponding bands. Data were representative of three independent experiments. (**c**) In the epidermis of stage 25 *Xenopus* embryos, FLNA labelling (in green, **c**1) is apically enriched in acetylated tubulin-positive MCCs (in magenta, **c**2,3). (**d**) Model illustrating the roles of miR-449 and other interconnected actors in MCC differentiation. Early cues required to trigger MCC differentiation involve the inhibition of BMP and Notch pathway. The expression of Multicilin (MCIDAS), CCNO and miR-449 is controlled by the Notch pathway activity. Notch repression is associated with the increase in MCIDAS, CCNO and miR-449 expression. Then, miR-449 miRNAs repress the Notch pathway inducing a double-negative feedback loop increasing miR-449 expression. MiR-449 inhibit cell cycle-related genes to stop proliferation and promote entry in differentiation. In addition, MCIDAS drives expression of centriole multiplication-related genes including CCNO, which then participates to centriole assembly and amplification. MCIDAS also contributes to increase FOXJ1 expression, which in turn controls ciliogenesis-related genes and apical actin remodelling through a RhoA-dependent mechanism. In parallel, miR-449 also control apical actin network remodelling by repressing R-Ras, promoting FLNA redistribution and modulating RhoA activity. Finally, miR-449 favour basal body maturation and anchoring by downregulating CP110. All these events are key pre-requisites for axoneme elongation and motile cilia formation during MCC differentiation. Plain lines indicate direct interactions; dotted lines identify pathways that may or may not be direct.

As observed for miR-449 morphants with rhotekin staining in *Xenopus* epidermis (Fig. 3b), PO-*rras*-injected MCCs displayed reduced actin cap and motile cilia staining, but maintained RhoA activation at the apical surface (Fig. 7c,e). This suggests that *RRAS* may interact with downstream effectors of RhoA during actin cap formation. In particular, previous works described an interaction between R-Ras and Filamin A (FLNA), a non-muscle F-actin cross-linking protein involved in epithelial cell shape, actin cytoskeleton remodelling and primary cilia formation^36^,^37^,^64^,^65^,^66^. FLNA can interact with the RhoA and Shroom3 signalling pathways^67^,^68^,^69^, and has also been involved in ciliogenesis and basal body positioning through its interaction with Meckelin^64^. We detected in control fully differentiated HAECs (Fig. 8a) FLNA in MCCs near apically docked basal bodies. By contrast, it was more homogeneously distributed in non-ciliated cells. The same apical enrichment of FLNA was observed in *Xenopus* MCCs (Fig. 8c). We finally noticed a miR-449-dependent subcellular redistribution of FLNA, after quantifying R-Ras and FLNA protein levels in different cellular fractions of proliferating HAECs that overexpress or not miR-449. In the absence of miR-449, R-Ras and FLNA were enriched in the membrane fraction (Fig. 8b). Following miR-449 overexpression, R-Ras disappeared from the membrane fraction, while FLNA was redistributed from the membrane to the cytoskeletal fraction (Fig. 8b). In a tentative model (Fig. 8d), we propose that the silencing of R-Ras by miR-34/449 in MCCs affects the interaction between R-Ras and FLNA, and favours a redistribution of FLNA in a cytoskeletal components involved into the anchoring of basal bodies. This mechanism would contribute to apical actin reorganization and basal body docking in MCCs.

In conclusion, our data further document how the miR-34/449 family can participate to multiciliogenesis through the repression of several important targets. MiR-34/449 may initially downregulate the expression of several cell cycle-regulated genes and members of the Notch pathway to promote entry into differentiation. Interestingly, miR-449 appears to be negatively regulated by Notch activity, supporting the existence of a double-negative feedback loop. At later time points of differentiation, this regulatory loop drives the accumulation of miR-449 at sufficient levels to downregulate additional targets implicated in more downstream events, such as CP110, involved in basal body maturation^48^, and R-Ras, shown here to be important for actin network assembly. From a wider point of view, this study illustrates well how a single miRNA family can contribute to complex cellular processes through its action on multiple targets belonging to different signalling pathways.

## Methods

### Subjects/tissue samples

Inferior turbinates or nasal polyps were from patients who underwent surgical intervention for nasal obstruction or septoplasty (kindly provided by Professor Castillo, Pasteur Hospital, Nice, France, or by Epithelix Sàrl, Geneva, Switzerland). The use of human tissues was authorized by the bioethical law 94–654 of the French Public Health Code after written consent from the patients.

### Isolation and culture of HAECs

Primary HAECs were dissociated and seeded on porous polyester membranes (5 × 10^4^ cells per membrane), in cell culture inserts (Transwell-clear, 0.33 cm^2^, 0.4 μm pores; Corning, Acton, MA) coated with human placenta collagen (0.2 mg ml^−1^; Sigma-Aldrich). HAECs were cultured in liquid–liquid conditions in the proliferation medium until confluency (5 days). Then, the culture medium was removed from the upper compartment and the airway epithelium was allowed to differentiate by using the differentiation medium consisting of 1:1 DMEM (Invitrogen, Gibco) and bronchial epithelial basal medium (Lonza) with the Clonetics complements for human Epidermal Growth Factor (hEGF) (0.5 ng ml^−1^), epinephrine (5 g ml^−1^), bovine pituitary extract (BPE) (0.13 mg ml^−1^), hydrocortisone (0.5 g ml^−1^), insulin (5 g ml^−1^), triio-dothyronine (6.5 g ml^−1^) and transferrin (0.5 g ml^−1^), supplemented with 200 UI ml^−1^ penicillin, 200 g ml^−1^ streptomycin and 0.1 nM retinoic acid (Sigma-Aldrich) in the basal compartment^9^. We analysed HAEC differentiation at four time points where Pr, Po, EC and LC represent the proliferating step at day 0, the polarization step at day 7, the early multiciliogenesis step at day 14 and the late multiciliogenesis step at day 21, respectively. Day number corresponds to the number of days after setting up the cells at an ALI.

### *Xenopus* injections

Eggs obtained from NASCO females were fertilized *in vitro*, dejellied in 2% cystein hydrochloride (pH 8.0) and cultured in modified Barth's saline, and injected as described^9^. cRNAs were generated with the Ambion mMessage mMachine kit (Life Technologies). pCS105/mGFP-CAAX (a gift from C. Chang, University of Alabama at Birmingham, USA) was linearized with AseI and cRNA was synthesized with Sp6 polymerase^9^. mRNAs (rhotekin, rGBD-GFP^56^, NICD and mRFP) were generated with the Ambion mMessage mMachine kit (Life Technologies). All injections were done at least twice using 0.25 ng of each mRNA.

### Immunocytochemistry

*Human*. Primary cultures of HAECs at EC and LC stages were used for detection of MCCs. Cells were fixed (4% paraformaldehyde, 15 min, 4 °C), rinsed (PBS–glycine 0.1 M, 10 min) and permeabilized (0.1% Triton X-100, 5 min). Only for γ-tubulin immunostaining to visualize centrioles and basal bodies, cells were fixed with methanol (10 min, −20 °C). Proliferating A549 airway epithelial cells grown on glass coverslip were used for detection of focal adhesion using anti-paxillin antibodies. Fixed cells were blocked for 1 h in 3% BSA and incubated for 1 h at room temperature or overnight at 4 °C with the appropriate primary antibodies (see Supplementary Table 1). Then, cells were incubated for 1 h with the appropriate secondary antibodies (Alexa Fluor, 1:500, Invitrogen), nuclei were stained with 4,6-diamidino-2-phenylindole (300 nM, Invitrogen) and, when indicated, F-actin was stained with Alexa Fluor 594 Phalloidin (1 U per staining). Stained cells were mounted with ProLong Gold antifade reagent (Invitrogen, Life Technologies)^9^.

*Xenopus*. For F-actin staining, embryos were fixed in 4% formaldehyde/PBT 1 h at 4 °C and stained with phalloidin-Alexa Fluor 555 (Invitrogen, 1:40) for 4 h at room temperature. For immunostaining, embryos were fixed in MEMFA (0.5 M MOPS, pH 7.4, 100 mM EGTA, 1 mM MgSO_4_, 3.7% formaldehyde). Whole-mount embryos or sections were blocked in 15% goat serum. The following primary antibodies were used: mouse anti-acetylated-tubulin (Sigma, 1:500), rabbit anti-RFP (Rockland, 1:500) and chicken anti-green fluorescent protein (GFP) (Aves, 1:500). After washing in PBT, sections or whole-mount embryos were incubated with the appropriate secondary antibody: anti-chicken Alexa Fluor 488 (1:500, Invitrogen), anti-mouse Alexa Fluor 555 (1:500, Invitrogen) or anti-mouse Alexa Fluor 647 (all from Invitrogen, 1:500). Epidermis fragments were peeled from embryos at stages 20 or 25 and mounted on a glass coverslip with fluoromount (Diagnostic BioSystem).

### Western blot experiments

Primary HAECs cells were harvested by scraping in Ripa lysis Buffer (Thermo Scientific Pierce) and cleared by centrifugation. Protein concentration was determined using the BCA assay (Thermo Fisher Scientific) and equivalent amounts of protein were resolved on SDS– polyacrylamide gels using Novex NuPAGE SDS–PAGE Gel System following the manufacturer's instructions. Proteins were transferred to polyvinylidene difluoride membranes and analysed by immunoblotting with appropriate primary antibodies and horseradish peroxidase (HRP)-conjugated secondary antibodies (Dako) according to the manufacturer's instructions. Immunoreactive bands were detected using immobilon ECL kit (Merck Millipore) on a LAS-3000 imager (Fujifilm). For subcellular protein fractionation, proliferating HAECs were trypsinized and centrifuged. Cell pellets were washed with ice-cold PBS and then treated with different buffers according to the manufacturer's instructions (Subcellular Protein Fractionation Kit for Cultured Cells, Thermo Scientific).

MO-*rras*-injected, PO-*rras*-injected or control neurula stage (st.19) *Xenopus laevis* embryos were lysed in Halt Protease Inhibitor Single Use Cocktail (Thermo Scientific), the lysate was cleared by centrifugation, protein concentration was determined by NanoDrop reading and identical amounts of protein for each condition were resolved on 12% SDS–polyacrylamide gel using the Hoefer Gel Caster system. Proteins were transferred to polyvinylidene difluoride membrane and analysed by immunoblotting with anti-rabbit R-Ras (1:300, Antibody Verify) or anti-mouse-α-tubulin (1:2,000, Sigma-Aldrich) primary antibody and HRP-conjugated secondary antibodies (1:2,000, Jackson). Immunoreactive bands were detected using Pierce ECL2 kit (Thermo Scientific) on Amersham Hyperfilm (GE Healthcare).

Uncropped scans of the most important blots were shown in Supplementary Fig. 5.

### Small GTPases activity assay

The activation of RhoA and Rac1, 2 and 3 were quantified using the glutathione *S*-transferase (GST) pulldown with recombinant proteins GST-Rhotekin-RBD and GST-PAK-PBD (Merck Milipore) that stoichiometrically interacts with GTP-bound Rho/Rac, as well as using the ELISA/G-LISA kits according to the manufacturer's instructions (Cytoskeleton, Denver, USA). The Rho activator, calpeptin, was provided by Cytoskeleton and the Rock inhibitor (Y-27632) was provided by Abcam PLC (Biotech Life sciences, UK).

HAECs were seeded on type-I collagen-coated surface and were transfected with miRNA mimics and siRNA. After 72 h, cells were lysed in cell lysis buffer containing proteinase inhibitor cocktail (Cytoskeleton), lysate were cleared by centrifugation and immediately quantified using Precision Red Advanced Protein Assay Reagent (Cytoskeleton). Approximately 400 μg of total proteins were incubated with the GST fusion protein at 4 °C for 30 min. Supernatants were then incubated with Pierce Glutathione Magnetic Beads for 30 min at 4 °C and washed three times with wash buffer (25 mM Tris pH 7.5, 30 mM MgCl_2_, 40 mM NaCl) using a magnetic rack. Input and pull-down samples were resuspended in loading buffer and blotted using Novex NuPAGE SDS–PAGE Gel System following the manufacturer's instructions.

In *Xenopus* MCCs, RhoA activation was revealed through the injection of RNA encoding the RhoA sensor rotekin rGBD-GFP (see above).

### Total RNA extraction

*Human*. Automated total RNA extraction was performed using QIAcube and miRNeasy kit from Qiagen, according to the manufacturer's instructions. Total RNAs were quantified using NanoDrop 1000 Spectrophotometer (Thermo Scientific) and integrity of samples (RNA Integrity Number (RIN)>8) was evaluated using RNA nano-chips on the Agilent 2100 Bioanalyzer Instrument (Agilent Technologies).

*Xenopus*. Total RNAs were isolated from animal caps dissected at stages 10–11 and cultured in modified Barth's saline (880 mM NaCl, 10 mM KCl, 8.2 mM MgSO_4_, 24 mM NaHCO_3_, 100 mM Hepes pH 7.4, 4.1 mM CaCl_2_, 3.3 mM Ca(NO_3_)_2_). Twenty explants for each sample (stages 14 or 25) were collected for RNA extraction. Total RNAs were isolated using the RNAeasy mini kit (Qiagen), according to the manufacturer's instructions, and quantified using a NanoDrop Spectrophotometer. Complementary DNAs were synthesized using iScript Reverse Transcription Supermix (BioRad).

### Quantitative RT–PCR

*Human*. qRT–PCR was performed using TaqMan Gene Expression Assay and TaqMan MicroRNA Assay (Life Technologies) on a Lightcycler 480 (Roche) according to the manufacturer's instructions. Expression levels of mature miRNAs and mRNA were calculated using the 2-ΔCT method, using respectively RNU44 and UBC as endogenous controls.

*Xenopus*. Primers were designed using Primer-BLAST Software. PCR reactions were carried out using SYBRGreen on a CFX Biorad qPCR cycler. All experiments were repeated at least twice on separate injections and the qRT–PCR was performed in triplicate. The relative expression of *RRAS* was normalized to the expression of the housekeeping gene *ornithine decarboxylase*. The qRT–PCR *RRAS* primers are as follows: forward: 5′- *gtaaccaaagaggaagcgctca* -3′; reverse: 5′- *ggatgacacaagggcaactttt* -3′.

### MiR-449 silencing and target protection experiments

*Human*. 3′-Cholesterol linked 2′-*O*-methyl miR-449a/b or miR-34b/c antisense oligonucleotide (antagomiR, antago-449: 5′- *c*_*s*_*u*_*s*_*c*_*s*_*uucaacacugccacau*_*s*_*u*_*s*_*u* **-Chol-3′, antago-34: 5′- *g*_*s*_*c*_*s*_*a*_*s*_*aucagcuaacuacacugc*_*s*_*c*_*s*_*u* -Chol-3′), Notch1 protector oligonucleotide 5′- a_s_a_s_a_s_aaggcaguguuucugug_s_u_s_a -Chol-3′ and *RRAS* protector oligonucleotide (PO-*RRAS*: 5′- *c*_*s*_*g*_*s*_*u*_*s*_*uggcagugacauuuauu*_*s*_*u*_*s*_*u* -Chol-3′) were purchased from Eurogentec (Seraing, Belgique). Phosphorothioate bonds are indicated by subscript ‘s'. The miR-449 antagomiR targets *Homo sapiens* miR-449a (full match) and miR-449b with one mismatch. The *RRAS* protector is a complementary antisense oligonucleotide targeting the conserved miR-449 binding site of the human *RRAS* 3′-UTR. The antagomiR or protector negative control was the Clear-miR (5′- *c*_*s*_*a*_*s*_*u*_*s*_*cgucgaucguagcg*_*s*_*c*_*s*_*a* -Chol-3′) from Eurogentec. AntagomiR or antisense protector (100 μM) were pre-incubated with FCS for 30 min at room temperature. Next, the antagomiR/FCS or protector/FCS mixture in differentiation medium (20 μM) were added to the apical side of primary HAECs. After 2 h at 37 °C, the apical medium was removed to restore the air–liquid interface. Transfection was repeated every 5 days with freshly prepared antagomiR or antisense protector, until control cells reached full differentiation (typically after 21 days)^9^.

*Xenopus*. Morpholino antisense oligonucleotides were as follows: MOs against miR-449 (GeneTools, LLC): miR-449a MO, 5′- *accagctaacattacactgcct* -3′; miR-449b MO, 5′- *gccagctaaaactacactgcct* -3′; miR-449c MO, 5′- *acagccagctagcaagtgcactgcc* -3′; control MO (MO-Neg), 5′- *tgcacgtttcaatacagaccgt* -3′. Ten nanograms of mixture of each miR-449 MO (449-MOs) was injected in one animal-ventral blastomere at the eight-cell stage. Protector MO directed against miR-449-binding sites in *rras* 3′-UTR (PO-*rras*): 5′- *gttggcaatgtaggtgcaattcgtt* -3′. PO-*rras* (5.7 or 7.5 ng) was injected in one animal-ventral blastomere at the eight-cell stage. MO blocking the translation of *rras* (MO-*rras*) has the following sequence: 5′- *gctccttggaactcatagtcgctgc* -3′. Fifteen or 25 ng of *rras* translation MO was injected in one animal-ventral blastomere at the eight-cell stage. The protector MOs directed against miR-449-binding sites in *Dll1* 3′-UTR have the following sequences: P1 MO: 5′- *cggcagtgcaacagtttatgtctgg* -3′; P2 MO: 5′- *aggcagtgactgtctgtagtttagc* -3′.

### Ectopic expression of miRNAs/siRNAs

Cells were grown to 30% confluency in proliferation medium on plastic, glass covserslip or on Transwell filters. Cells were then transfected with synthetic negative control miRNA (miR-Neg, Ambion) or synthetic miR-449a/b miRNAs (Ambion) (10 nM final concentration). Total RNAs or proteins were extracted, or immunostaining was performed, from 24 to 72 h later. For siRNA experiments in differentiating HAECs or A549 cell line, cells were transfected with a siRNA against the human *RRAS* (si-*RRAS*), *ARHGDIB* (si-*ARHGDIB*) or *ARHGAP1* (si-*ARHGAP1*) transcripts, or with a negative control siRNA (si-Neg) (Stealth RNA_i_ siRNAs, Life Technologies) (20 nM final concentration) using Lipofectamine RNAi Max Reagent (Invitrogen) in OPTIMEM (Invitrogen) according to the manufacturer's instructions. On the next day, an additional transfection was performed using the same procedure, before HAEC differentiation was induced in ALI, on the third day. Finally, HAECs were harvested for western blot analyses or processed for immunofluorescence experiments after 3 days for proliferating cells on glass coverslip or 7, 14 and 21 days for primary cultures in ALI.

### Plasmid constructs and luciferase measurements

Sequence from the wild-type or mutants 3′-UTR of *RRAS*, *ARHGDIB*, *ARHGAP1*, NDRG1 and DAAM1 were synthesized (gBlocks Gene Fragments, Integrated DNA Technologies) and cloned into psiCheck2 vector (Promega). For mutated 3′-UTRs, three bases of each seed region were changed by complementary bases. PsiCheck2 constructions were co-transfected with synthetic miRNA mimics (Ambion, Applied Biosystems) with or without antagomiRs or antisense protectors into HEK293T cells, and luciferase activity was measured using the dual reporter luciferase assay kit (Promega), according to the manufacturer's protocol.

### ISH on *Xenopus* embryos

Whole-mount ISH analysis was done as follows: embryos were fixed in MEMFA (0.1 M MOPS pH 7.4, 2 mM MgSO_4_, 1 mM EGTA, 3.7% v/v formaldehyde) overnight at 4 °C, dehydrated in 100% ethanol or methanol overnight at −20 °C, then rehydrated in PBT (PBS+0.5% Tween20), bleached for 5–10 min in H_2_O_2_, and hybridized overnight with the chosen probe. After hybridization, the embryos were washed at increasing stringency in SSC/10% CHAPS, rinsed extensively n MABX (Maleic Acid Buffered solution +0.5% Triton X), then incubated overnight at 4 °C under gentle agitation with the appropriate antibody in MABX, 2% Roche Blocking Reagent, 15% goat serum or FCS. On the final day, embryos were extensively washed in MABX, then stained with Roche BM-Purple^70^. *RRAS* digoxigenin-labelled sense and antisense riboprobes (4202704 (IRBH 18A04), IRHB XGC, Source BioScience Life Sciences) and fluorescein-labelled antisense α-tubulin riboprobe^17^ were generated from linearized plasmids using RNA-labelling mix (Roche). A locked nucleic acid antisense probe against the mature form of miR-449a was described previously^9^. For fluorescent ISH (FISH) on sections, embryos were fixed in MEMFA for 2 h at room temperature or overnight at 4 °C, stored in methanol at least 24 h at −20 °C, rehydrated and washed in triethanolamine (0.1 M)/acetic anhydrid. Embryos were then transferred in successive sucrose washes from 5% to 30% sucrose in PBS+Tween 20 (0.1% v/v).They were then embedded in O.C.T. Compound (VWR Chemicals Prolabo), flash frozen and 12-μm-thick sections were prepared with a CM3050S Leica cryostat. Slides were kept at −80 °C overnight. FISH analysis was then carried out using Tyramide Signal Amplification—TSA TM Plus Cyanine 3/Fluorescein System (PerkinElmer). Before hybridization, and after Proteinase K digestion (3 min at 2 μg ml^−1^), endogenous peroxydase activity was blocked in a bath of H_2_O_2_ 3% in PBS for 20 min. Sections were hybridized with a mixture of digoxigenin- and fluorescein-labelled probes 40 ng each) overnight at 60 °C. Following washes (two times with 2% SSC/0.1% CHAPS at 60 °C; two times with 0.2% SSC/0.1% CHAPS at 37 °C), the digoxigenin-labelled probe was revealed first through incubation with a mouse anti-DIG antibody conjugated to HRP (POD) (Roche, 1:500), followed by incubation in Cy3 fluorophore amplification reagent (1/50 in the TSA diluent during 10 min). This reaction was then blocked in a bath of 2% H_2_O_2_ for 20 min. Next, the fluorescein-labelled probe (*rras*, *arhgdib* or *arhgp1*) was revealed with a mouse anti-fluorescein POD-conjugated antibody (Roche, 1:500), followed by incubation in Cy5 fluorophore amplification reagent (1/50 in the TSA diluent during 10 min). This second reaction was blocked in a bath of 2% H_2_O_2_ for 20 min. Following double FISH analysis, immunostaining with anti-GFP antibody was performed and slides were processed for confocal imaging. *Xenopus* embryos at stage 34 were fixed in paraformaldehyde 4% overnight and frozen in 100% methanol at −20 °C overnight before performing ISH with digoxigenin-labelled locked nucleic acid (Exiquon) probes against miR34b (5′*-DIG*- *caatcagctaactacactgcctg* *-DIG*-3′)^9^.

### Confocal microscopy

*Human*. Images were acquired using the Olympus Fv10i or the Leica SP5 confocal imaging systems.

*Xenopus*. Flat-mounted epidermal explants were examined with a Zeiss LSM 780 confocal microscope.

Three-colour confocal *z*-series images were acquired using sequential laser excitation, converted into single plane projection and analysed using ImageJ software.

In Supplementary Table 1 is provided a list of the antibodies used in our study.

## Additional information

**How to cite this article:** Chevalier, B. *et al*. miR-34/449 control apical actin network formation during multiciliogenesis through small GTPase pathways. *Nat. Commun.* 6:8386 doi: 10.1038/ncomms9386 (2015).

## Supplementary Material

Supplementary Information Supplementary Figures 1-5 and Supplementary Table 1
