# Supplementary material for: miR-34/449 control apical actin network formation during multiciliogenesis through small GTPase pathways
Source: Nat Commun. 2015 Sep 18;6:8386. doi: 10.1038/ncomms9386 (PMC4595761; doi:10.1038/ncomms9386)
Supplement: Supplementary Information — Supplementary Figures 1-5 and Supplementary Table 1 [file ncomms9386-s1.pdf]

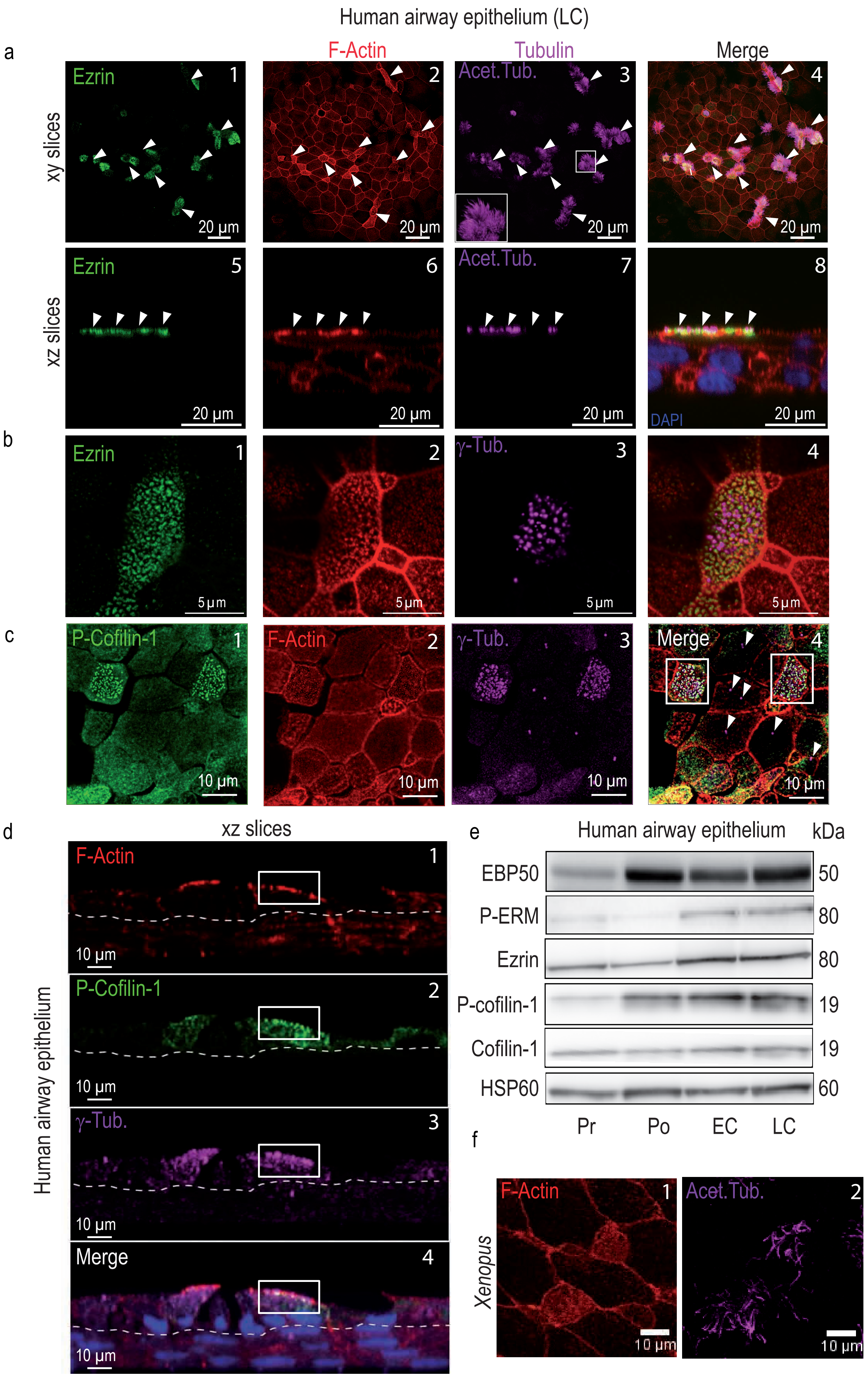

Supplementary Figure 1

Supplementary Figure 1. Apical actin meshwork reorganization in vertebrate MCCs. (a) Differentiated HAECs at LC stage were stained for ezrin (a1,5), F-actin (a2,6) and motile cilia (a3,7). Nuclei were stained with DAPI (a6). Panels a5-8 are orthogonal views (xz slices) of panels a1-4. In acetylated tubulin-positive MCCs denoted with white arrowheads and white framework (a3,7), F-actin is apically enriched (a2, a6) and colocalizes with ezrin staining (a1,5 and a4, 8). (b) γ-tubulin-positive basal bodies (in magenta, b3,4) colocalize with ezrin (b1) and apical F-actin (b2). (c) Differentiated HAECs at LC stage were stained for P-cofilin-1 (in green, c1,4), F-actin (in red, c2, 4) and γ-tubulin-positive basal bodies (in magenta, c3,4). P-cofilin1 co-localizes with the apical actin meshwork near basal bodies in MCCs (white framework in c4). No P-cofilin-1 signal is observed near centrioles in non-ciliated cells (white arrowheads, c4). Panels d1-4 are orthogonal views (xz slices) of differentiated HAECs at LC stage illustrating that P-cofilin-1 is enriched with the apical actin meshwork and near basal bodies in MCCs (white framework, d1-4). (e) Dynamics of the phosphorylation state of ezrin or ERM and cofilin1 and of EBP50 expression during HAEC differentiation are indicative of actin remodeling (Pr: proliferating HAECs; Po: polarization stage, ALI days 5-12; EC: early ciliogenesis, ALI days 14-21; LC: late ciliogenesis, from ALI days 28). HSP60 was used as a loading control. (f) In the epidermis of stage 25 *Xenopus* embryos, F-Actin (in red, f1) is apically enriched in acetylated tubulin-positive MCCs (in magenta, f2).

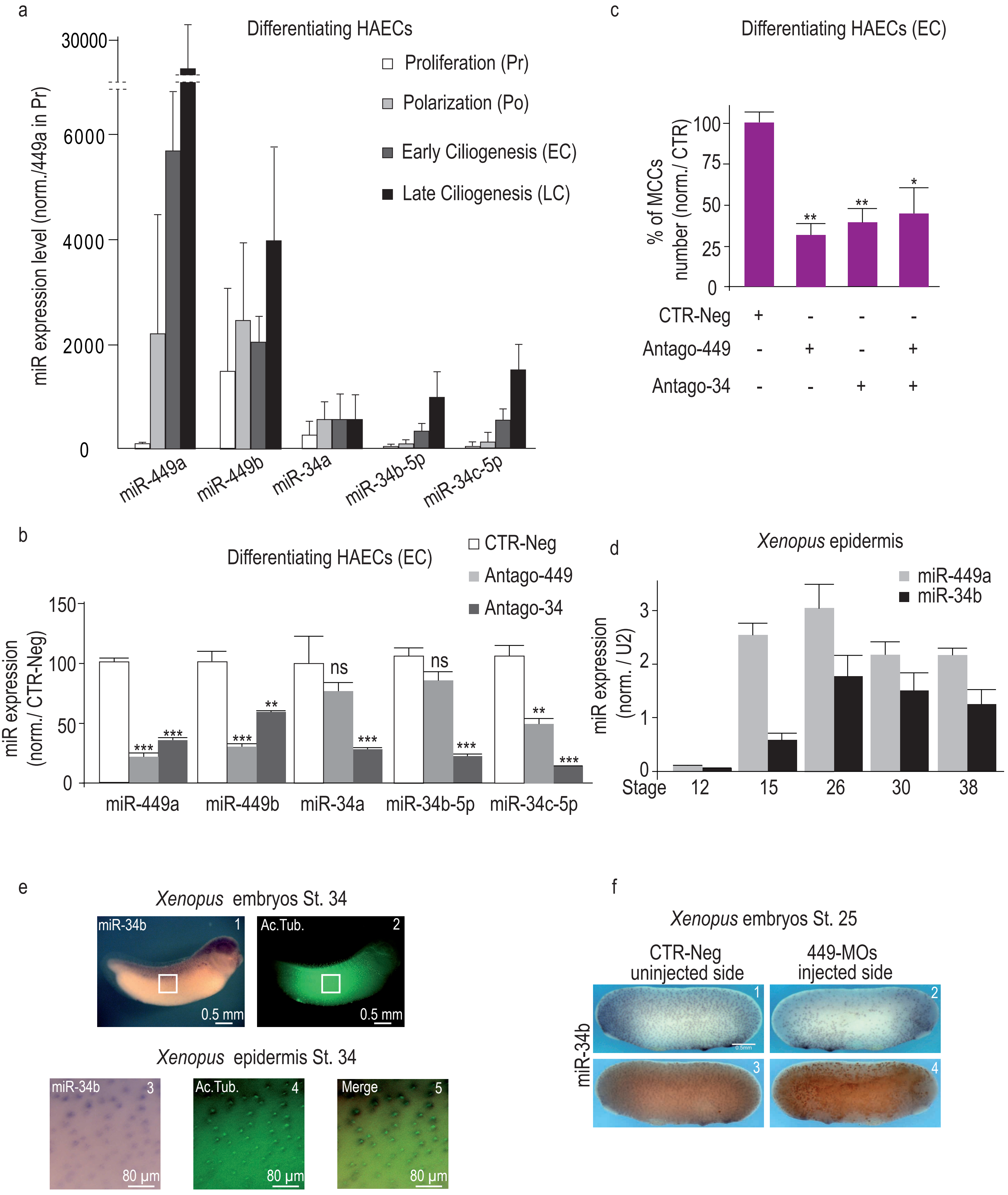

Supplementary Figure 2

Supplementary Figure 2. Role of miR-34 and miR-449 during multiciliogenesis in HAECs and *Xenopus* epidermis. (a) Expression level of miR-449a (1), miR-449b (2), miR-34a (3), miR-34b-5p (4) and miR-34c-5p (5) was measured by real-time PCR at the four stages of HAEC differentiation (Pr, Po, EC and LC). MiRNA levels are normalized with *RNU44* and normalized to miR-449a level in Pr stage. Data represent the mean and s.d. of 3 independent experiments. (b,c) Effect of a treatment by negative control antagomiR (CTR-Neg), anti-miR-449a/b (Antago-449), anti-miR-34 (Antago-34) or an equal mixture of Antago34/449 on differentiating HAECs at EC stage. (b) The effects of Antago34 and Antago-449 versus CTR-Neg on miR-34/449 expression on differentiating HAECs at EC stage were evaluated by real-time PCR. MiRNA levels are normalized with *RNU44* and normalized to miR-449a level at 100% in CTR-Neg condition. Data represent the mean and s.d. of 3 independent experiments (***, $p<0.001$; **, $p<0.01$; *, $p<0.05$; ns : not significant; Student's t-test). (c) The histogram indicates the average percentage of MCC cell number (magenta) relative to control (means ± s.d. from 3 donors, **, $p<0.01$; *, $p<0.05$; Student's t-test). (d) Expression level of miR-449a and miR-34b (the two major members of the miR-34/449 family expressed in the *Xenopus* embryonic epidermis (GEO, GSE22147) at different developmental stages of *Xenopus* embryos. MiRNA levels are normalized with U2. Data represent the mean and s.d. of 3 independent experiments. (e) *In situ hybridization* of miR-34b (e1, 3) together with co-staining of MCCs with acetylated tubulin antibodies (e 2, 4) indicated that miR-34b is expressed in acetylated-positive MCCs in *Xenopus* embryonic epidermis and nephrostomes at stage 34. Panels e3 and e4 are enlarged views of area bounded by a white rectangle in panels e1 and e2, respectively. Panel e5 is a merged image of panels e3 and e4. (f) 449-MOs also suppress miR34b expression. 8 cell-stage *Xenopus* embryos were injected in the epidermis precursor blastomeres with 449-MOs and FLDx (in orange/brown, f3-4). *In situ hybridization* revealed that miR34b is suppressed in injected side of miR449 morphants embryos in 93% of cases whereas miR-34b is still expressed in negative control uninjected side.

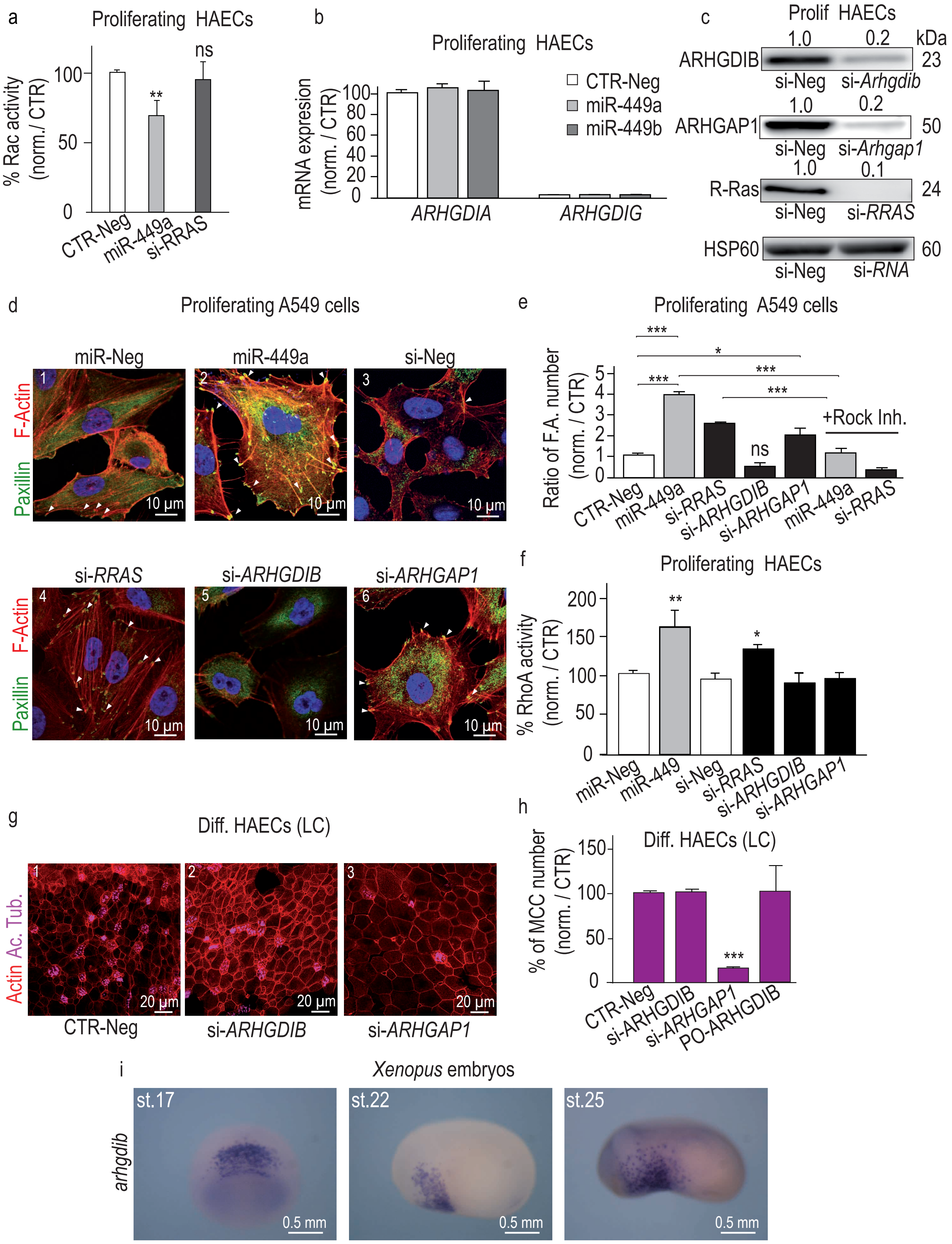

Supplementary Figure 3

Supplementary Figure 3. Effects of miR-449, *ARHGAP1*, *ARHGDIB* or *RRAS* silencing on actin web or RhoA/Rac activity in proliferating lung epithelial cells. (a) Rac activity was measured in proliferating HAECs in response to miR-449a or si-*RRAS*. The histogram indicates Rac activation normalized to the control (CTR-Neg) set to 100%. (b) Transcript expression levels of *ARHGDIA and ARHGDIG* were analyzed using real-time RT-PCR following miR-449a/b overexpression (48h) in proliferating HAECs and normalized with *UBC* transcript as an internal control. (c) Proliferating HAECs were transfected with small interfering RNA (si-RNA) against transcripts of *ARHGAP1* (si-*ARHGAP1*), *ARHGDIB* (si-*ARHGDIB*) or *RRAS* (si-*RRAS*) versus negative control (si-Neg). Protein levels were normalized with HSP60 as an internal control. Quantification of protein levels are indicated above each corresponding band and are representative of at least three independent experiments. (d-f) Proliferating HAECs or A549 cells were transfected for 72h with miR-Neg (d1), miR-449a (d2), si-Neg (d3), si-*RRAS* (d4), si-*ARHGDIB* (d5) or si-*ARHGAP1* (d6) in absence (d) or presence (e) of a Rock inhibitor (Y27632, 10 µM) to examine RhoA implication. Then, cells were stained for F-Actin (red), focal adhesions (anti-paxillin in green) and nuclei (DAPI in blue) (d). (e) The focal adhesion number per cell was quantified and normalized to control. (f) RhoA activity was then measured in each condition in proliferating HAECs. The histogram indicates RhoA activation normalized to the control set to 100%. (g) Differentiating HAECs were transfected at seeding time with si-Neg (g1), si-*ARHGDIB* (g2) or si-*ARHGAP1* (g3) and F-actin (in red) and motile cilia (in magenta) were stained. (h) The histogram indicates the number of MCCs normalized in percentage from control (CTR-Neg) in HAECs at LC stage. All data are means ± s.d. from at least three independent experiments (***, $p<0.001$, Student's t-test). (i) *In situ* hybridization of *arhgdib* mRNAs on whole-mount *Xenopus* embryos at stages 17, 22 and 25, showing no expression of *arhgdib* in *Xenopus* epidermis. Staining was detectable only in blood cells.

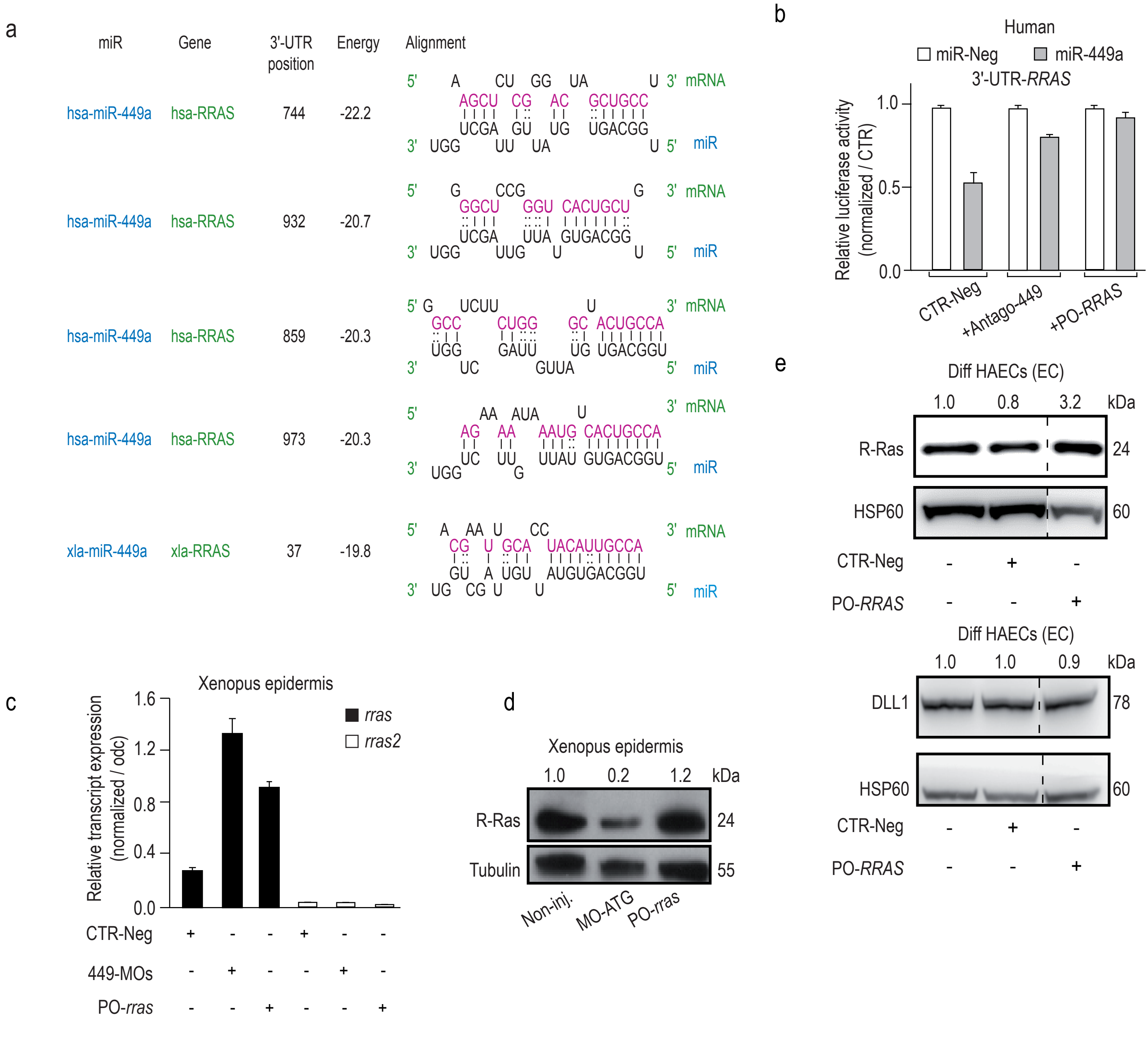

Supplementary Figure 4

Supplementary Figure 4. (a) **MiR-449 binding sites located in the 3'-UTR of *Homo sapiens* (hsa) or *Xenopus* laevis (xla) *RRAS* mRNA were identified *in silico* using "microcible" miRNA target prediction tool available on our laboratory website (**http://www.genomique.info:8080/merge/index**).** (b) Inhibition of miR-449 with Antago-449 or miR-449::*RRAS* protector oligonucleotides (PO-*RRAS*) specifically prevent miR-449 binding on *RRAS* 3'-UTR. miR-449a transfection in HEK293 cells strongly reduces relative luciferase activity of wild-type 3'-UTR chimeric constructs of *RRAS*. This effect is strongly blocked by antago-449a/b or miR-449::*RRAS* protector co-transfection, whereas it is not significantly affected by co-transfection with negative antagomiR or protector controls (CTR-Neg). Values were normalized to the internal *Renilla* Luciferase control. (c) Real-time RT-PCR expression of *rras* or *rras2* in *Xenopus* embryos injected with control MO (CTR-Neg), PO-*rras*, or 449-MOs. Unlike *rras*, *rras2* expression was very low and was not affected in either 449-MOs or PO-*rras*, indicating that only *rras* is a true miR-449 target in *Xenopus*. Transcripts levels were normalized against *Odc* transcript as an internal control. Error bars denotes standard deviation from three independent experiments. (d) Protecting *rras* mRNA against interaction with miR-449 resulted in increased levels of R-Ras protein in *Xenopus*. R-Ras levels in embryos injected with PO-*rras* were about 20% higher than in control non-injected embryos. By comparison, blocking *rras* translation by injection of MO-ATG-*rras* led to an 80% decrease in the amount of R-Ras. Embryos were injected at 1 cell stage and lysed at stage 19. Signal intensity was measured with ImageJ, using α-tubulin as an internal control. Data are representative of three independent experiments. (e) In differentiating HAECs at stage EC, protecting human *RRAS* mRNA against interaction with miR-449 (PO-*RRAS*) resulted in increased levels of R-Ras protein in comparison to treatment with negative control (CTR-Neg) but not of DLL1 protein, indicating the specificity of PO-*RRAS* and that *RRAS* is an effective miR-449 target in human. Protein levels were normalized with HSP60 as an internal control. Experiments were representative of three donors.

Figure 1e

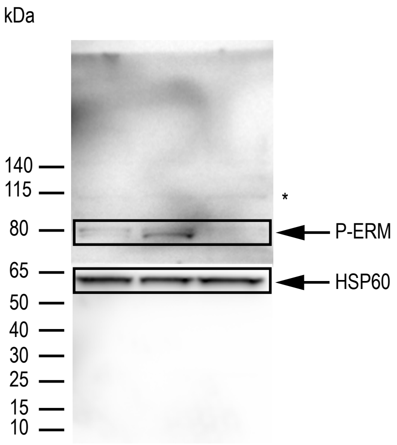

Figure 4d

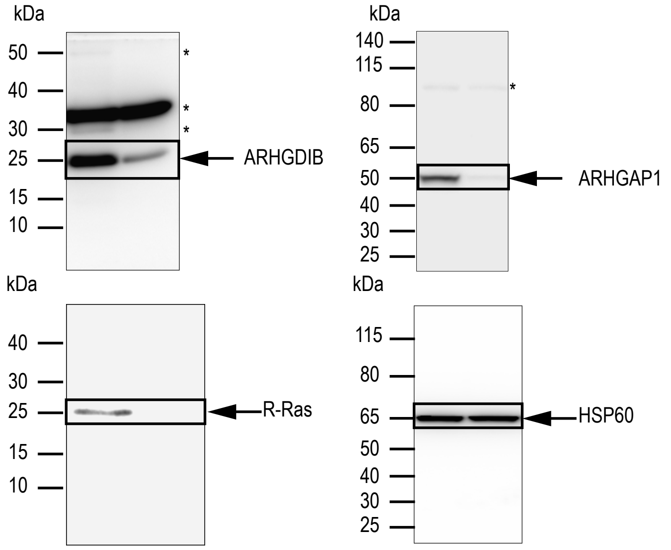

Figure 5d

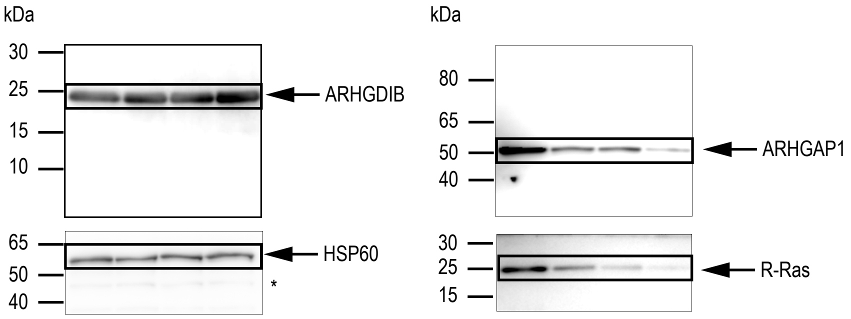

Figure 8b

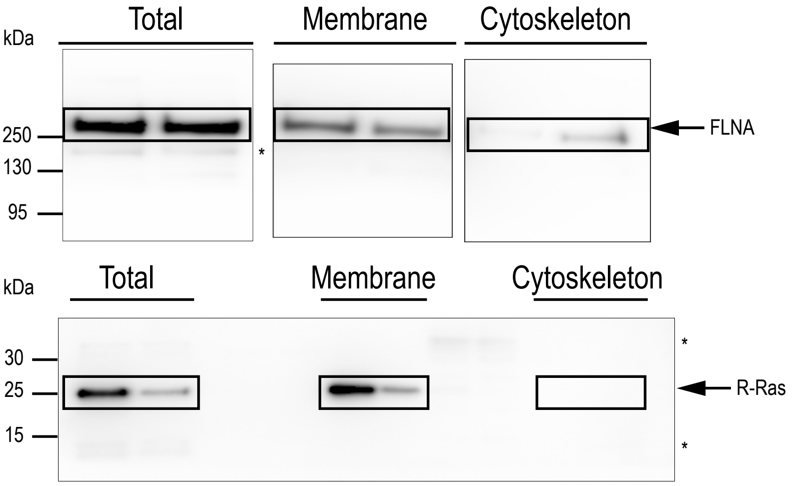

Supplementary Figure 1e

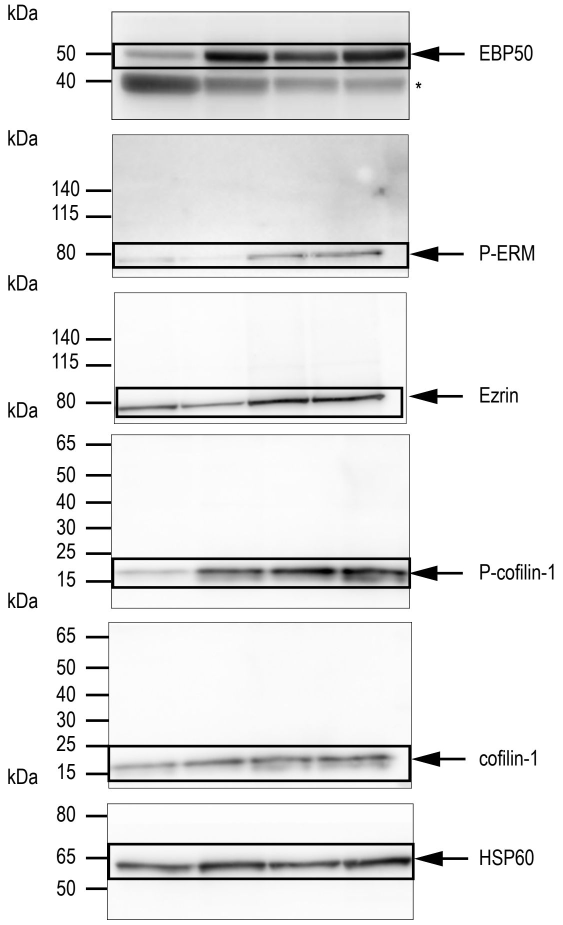

Supplementary Figure 3c

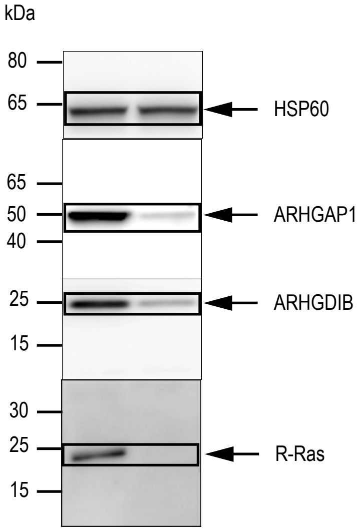

Supplementary Figure 4d

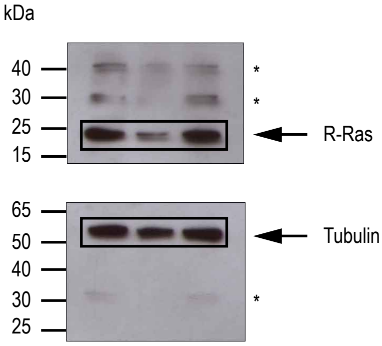

Supplementary Figure 4e

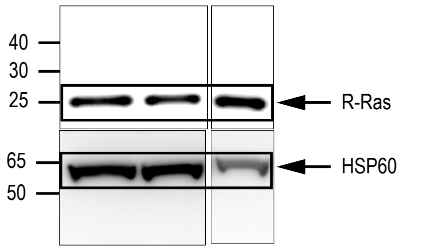

Supplementary Figure 5. Full scans of Western data

* Non specific band

Supplementary table 1. List of Antibodies used :

Sp.	Target	Réf./clone	Manufacturer	Dilution
Ms	Acetylated-Tubulin	6-11B-1	Sigma-Aldrich	1/1000 (IF)
Ms	γ-Tubulin	GTU-88	Sigma-Aldrich	1/1000 (IF)
Ms	CD151	14A2.H1	BD biosciences	1/100 (IF)
Rb	R-Ras	C-19	Santa Cruz Biotechnology, Inc.	1/1000 (WB),<br>1/400 (IF)
Rb	Ezrin	07-130	Merck Millipore	1/1000 (WB),<br>1/100 (IF)
Rb	P-ERM	41A3	Cell Signaling Technology	1/1000 (WB),<br>1/100 (IF)
Gt	Actin	I-19	Santa Cruz Biotechnology, Inc.	1/5000 (WB)
Rb	ARHGAP-1	H-76	Santa Cruz Biotechnology, Inc	1/500 (IF)
Rb	ARHGDIB/RHOGDI2	Ab15198	ABCAM	1/500 (IF)
Ms	Paxillin	349/Paxillin	BD Biosciences	1/10000 (IF)
gt	HSP60		Santa Cruz Biotechnology, Inc.	1/5000 (WB)
Rb	RhoA	67B9	Cell Signaling Technology	1/1000 (WB)
Rb	P-Cofilin 1	(hSer3)-R	Santa Cruz Biotechnology, Inc.	1/500 (WB)
Rb	Cofilin	3312	Cell Signaling Technology	1/1000 (WB)
Rb	Filamin A	HPA002925	Sigma-Aldrich	1/400 (IF),<br>1/1000 (WB)
Gt	DLL1	Ab85346	ABCAM	1/500 (WB)
HRP conjugate anti-Ms/Rb/Gt			Dako	Lot Dependent
